# Genetic modulation of the iris transillumination defect: a systems genetics analysis using the expanded family of BXD glaucoma strains

**DOI:** 10.1111/pcmr.12106

**Published:** 2013-04-13

**Authors:** Shankar Swaminathan, Hong Lu, Robert W Williams, Lu Lu, Monica M Jablonski

**Affiliations:** 1 Department of Ophthalmology, Hamilton Eye Institute, University of Tennessee Health Science CenterMemphis, TN, USA; 2 The Affiliated Hospital of Nantong UniversityNantong, China; 3 Department of Anatomy and Neurobiology, University of Tennessee Health Science CenterMemphis, TN, USA; 4 Jiangsu Key Laboratory of Neuroregeneration, Nantong UniversityNantong, China

**Keywords:** iris transillumination defect, QTL analysis, systems genetics, epigenetics, mathematical modeling

## Abstract

We investigated the contributions of *Tyrp1* and *Gpnmb* to the iris transillumination defect (TID) in five age cohorts of BXD mice. Using systems genetics, we also evaluated the role of other known pigmentation genes (PGs). Mapping studies indicate that *Tyrp1* contributes to the phenotype at all ages, yet the TID maps to *Gpnmb* only in the oldest cohort. Composite interval mapping reveals secondary loci viz. *Oca2*, *Myo5a, Prkcz, and Zbtb20* that modulate the phenotype in the age groups up to 10–13 months. The contributions of *Tyrp1* and *Gpnmb* were highly significant in all age cohorts. Moreover, in young mice, all six gene candidates had substantial interactions in our model. Our model accounted for 71–88% of the explained variance of the TID phenotype across the age bins. These results demonstrate that along with *Tyrp1* and *Gpnmb*, *Oca2*, *Myo5a, Prkcz, and Zbtb20* modulate the TID in an age-dependent manner.

**Significance**

Iris pigmentation is a complex genetic trait controlled by a network of genes whose coordination and regulation are poorly understood. Pigmentation abnormalities are clinically significant; they can lead to albinism, iris transillumination defects (TIDs), and potentially pigment dispersion glaucoma. We demonstrate for the first time the simultaneous contribution of multiple genes to the TID phenotype. The outcome of our studies using age-specific cohorts to elucidate the impact and interactions of the known pigmentation genes and their control over the progression of TID is unparalleled. Identifying which genetic modifier(s) regulates the disease during its progression will be significant in formulating targeted therapeutic options.

## Introduction

Genetic variations in mice with regard to the color coat have had a significant influence on the discovery of genes influencing pigmentation (Bennett and Lamoreux, 2003) and on the evolution of mouse genetics (Paigen, 2003). Studies using these strains have been responsible in revealing a wide array of important genetic pathways related to pigmentation. Recently, many groups have reported various approaches to examine these important pigment-related pathways using the iris (Anderson et al., 2008; Brooks et al., 2007; Trantow et al., 2009, 2010). The mouse iris is unique and represents a powerful opportunity for studying pigment cell biology due to its dense pigmentation. The iris and iridial melanin play an important role in influencing vision (Iwata et al., 2000; Summers, 1996; Wong et al., 2005). Moreover, several diseases, including forms of oculocutaneous albinism, Horner's syndrome, Fuchs heterochromic iridocyclitis, Waardenburg syndrome, Hermansky–Pudlak syndrome, and pigment dispersion, involve the pigmentation of the iris. Despite its biological significance, there is a definite knowledge gap concerning the basic molecular and cellular processes influencing pigmentation of the iris. Thus, gone are the days, when iris had only cosmetic influence.

The iris is composed of three types of melanin-containing cells: neural crest-derived iris stromal melanocytes; neuroepithelium-derived iris pigment epithelial cells; and bone marrow-derived antigen-presenting cells of the iris stroma. Melanin is synthesized by melanocytes and iris pigment epithelial cells, while the antigen-presenting cells attain pigmentation via phagocytosis (Anderson et al., 2008; Fine and Yanoff, 1979; Rodrigues et al., 1982). Pigment-producing cells of the iris synthesize melanin within melanosomes utilizing many of the same well-known pathways akin to the melanocytes in skin and hair (Lin and Fisher, 2007). An important point for consideration is that the pigment-producing cells of the iris exhibit many unique properties (Hong et al., 2006; Hu, 2005; Li et al., 2006; Liu et al., 2005; Smith-Thomas et al., 2001; Ward and Simon, 2007; Wistow et al., 2002). For example, cultured murine iris melanocytes do not respond to a melanocyte-stimulating hormone, a potent mitogenic and melanogenic hormone to melanocytes cultured from skin (Li et al., 2006). These factors presumably reflect an adaptation or consequence of the genetic and environmental factors impacting various pigment-producing cells. Thus, studies of the iris provide an opportunity to reveal new molecular pathways governing these differences and represent a robust opportunity to obtain new insight into the factors influencing pigment cell biology iris (Anderson et al., 2008).

Pigment synthesis takes place in the melanosome, a lipid-bound organelle within melanocytes. As mouse strains with coat color variations harbor mutations that influence melanosomes on a whole, these mutations may also affect the iris and give rise to abnormal ocular phenotypes. However, there could also be differences in coat and the iris phenotypes, which could mainly be due to difference in the development of iris and the coat. Specifically, melanocytes influencing coat color are solely derived from neural crest, whereas pigment cells in iris are of diverse origin (neural crest-derived melanocytes of iris stroma, neuroepithelial-derived cells of the iris pigment epithelium and bone marrow-derived antigen-presenting cells). Genetic abnormalities in neural crest would thus affect both iris and coat, while that affecting neuroepithelial cells and bone marrow would only affect the iris, thereby leading to a difference in the phenotypes. (Anderson et al., 2008). The production of brown-black pigment or eumelanin is a multistep chemical reaction regulated by multiple gene products including *Tyrp1* (tyrosinase-related protein 1) and *Dct* (dopachrome delta-isomerase, tyrosine-related protein 2) (Hirobe and Abe, 2007; Matsunaga et al., 2002; Solano et al., 2000; Winder et al., 1994). Gene products such as *Oca2* (oculocutaneous albinism II) and *Myo5a* (myosin VA) have also been found to be critical for melanogenesis. The pigmentation pattern of the iris is a complex genetic trait controlled by an intricate network of genes whose coordination and regulation are poorly understood.

DBA/2J (D2) has served as the preeminent mouse model for pigment dispersion syndrome (PDS) (Anderson et al., 2001, 2008; Chang et al., 1999; Howell et al., 2007; John et al., 1998). Howell et al. have previously shown the D2 iris disease is genetically separable into two distinct traits, iris pigment dispersion (IPD) and iris stromal atrophy (ISA) (Chang et al., 1999; Howell et al., 2007). Iris pigment dispersion is characterized by a breakdown of the posterior iris pigment epithelium, slit-like transillumination, and prominent pigment dispersion. Iris pigment dispersion is caused by a mutation in the glycoprotein (transmembrane) nmb gene (*Gpnmb*R150X). They have also shown that ISA is characterized by deterioration of the iris stroma, and an accumulation of stromal pigment and cell debris in the drainage structures and is caused by a recessive mutation in the tyrosinase-related protein 1 gene (*Tyrp1b*) (Chang et al., 1999; Howell et al., 2007). In this study, we use pigment dispersion to indicate the phenotype characterized by any aberrant deposition of pigment throughout the anterior chamber of the eye. Clinically, pigment dispersion is a primary feature of PDS and pigment dispersion glaucoma and can also occur in pseudoexfoliation syndrome, intraocular melanoma, and uveitis (Anderson et al., 2008; Ball, 2004; Ritch et al., 1996; Shuba et al., 2007; Sowka, 2004). From a clinical perspective, the pigment sloughs off the posterior iris and blocks the drainage of aqueous humor through the trabecular meshwork. In terms of an acquired iris phenotype, clinically, it can be due to surgical complications wherein there is damage caused to the iris pigment epithelium leading to IPD or iris transillumination defect (TID).

John and colleagues have shown that the eyes of D2 mice have no obvious abnormalities at 3 months of age. However, by 6 months, iris depigmentation develops in a large percentage of D2 mice; by 8 months, an increase in IOP followed by damage to the optic nerve develops. Recombinant inbred (RI) strains of mice are a useful resource for identification of the genetic sources of variation in phenotype, in our case, the severity of TID (Geisert et al., 2009; Jablonski et al., 2011; Lu et al., 2011a,b). The largest panel of these strains, the BXD family, consists of the inbred progeny of a cross between wild-type normotensive C57BL/6J (B6 or B) that does not have any mutations with the glaucoma-prone D2 strain (digenic mutations in *Gpnmb* and *Tyrp1*). Collectively, these 80 BXD strains are among the largest RI sets, and they have been used extensively in genetic and genomic studies of the eye and central visual system (Geisert et al., 2009; Jablonski et al., 2011; Lu et al., 2011a,b). Digenic mutations in *Tyrp1* and *Gpnmb* cause pigmentary dispersion syndrome and pigmentary glaucoma in DBA/2J mice (Anderson et al., 2001). While no exonic mutations in either gene have been found in human glaucoma patients (Anderson et al., 2001; Lynch et al., 2002), given the data for mice, one would anticipate that only a ‘two-hit’ model would be required to reveal the coupling to disease in humans.

While several genes involved in pigmentation are known—*Tyrp1*, *Gpnmb*, *Tyr,* and *Dct*—it is not clear how they are genetically regulated or precisely how they function together (Lu et al., 2011a,b). Studies have shown that *Gpnmb* and *Tyrp1* mutations alter melanosomes, thereby allowing pigment production to occur normally, while cytotoxic intermediates of pigment production escape, inducing iris disease (Anderson et al., 2001). *Gpnmb* and *Tyrp1* proteins have known to contain several motifs common to melanosomal proteins, similar to tyrosinase and each other (Anderson et al., 2001). *Tyrp1* is reported to be the most abundant melanosomal glycoprotein (Tai et al., 1983). It influences melanosome structure (Moyer, 1966) and is required for the stabilization of a membrane-bound melanogenic protein complex (Kobayashi et al., 1998).

The parental strains from which the BXD recombinant inbred mice were generated—B6 and D2—vary in their degree of TID. B6 mice have no ocular aberrations, while D2 harbor mutations in *Tyrp1* and *Gpnmb* (Anderson et al., 2001; Chang et al., 1999; John et al., 1998), both of which are required for manifestations of severe and progressive TID. Because the BXD progeny segregate these and all other genes, the TID also segregates, thus providing a powerful genetic reference panel. While it has been previously reported that *Tyrp1* and *Gpnmb* contribute significantly to the TID phenotype (albeit they are not the only genes), we are confident that this study is the first of its kind to simultaneously evaluate the contribution of multiple pigmentation genes to the TID phenotype (Lu et al., 2011a). This study is unique also because of the separation of mice into five distinct age cohorts that allowed us to elucidate the impact of the known pigmentation genes as a function of age. Our objective in the endeavor was thus to investigate the relative contributions of *Tyrp1* and *Gpnmb* to the iris TID over the course of the disease (time cohort and disease progression) using our large family of BXD recombinant inbred mice and a large repository of sequence variation data and bioinformatics analysis tools available on http://GeneNetwork.org (http://www.genenetwork.org/webqtl/main.py). We also sought to evaluate the plausible role of other known pigmentation genes along with potential genetic interactions using systems genetics along with mathematical modeling.

## Results

### Development of iris transillumination defect grading scale

**Figure 1:**
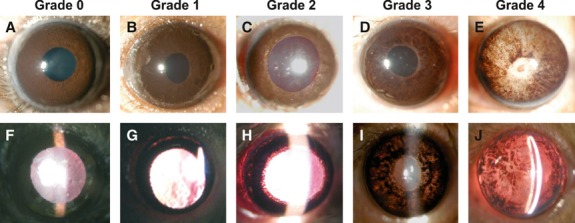
Appearance of anterior segment and iris transillumination defect (TID) of BXD RI mice by slit-lamp biomicroscopic examination. Within each column are representative images of TIDs corresponding to grades 0–4. (A–E) show broad beam illumination to document the overall degree of iris damage including pupillary rim defects and loss of pigmentation. (F–J) show transillumination defects obtained by shining a narrow beam of light into the pupil and documenting the pattern of light that shines through the body of the iris.

We categorized the level of TID into five grades encompassing the full range of the phenotype. Representative photographs of each grade are shown in Figure 1. A brief description of each grade category is as follows:

Grade 0: The iris appears intact when illuminated with a broad beam of light, and no light is transmitted through the iris when a narrow beam of light is shined through the pupil;
Grade 1: The iris is not visibly aberrant when illuminated with a broad beam of light, and a very small amount of light is transmitted through the iris, typically in the mid-peripheral region of some areas, when a narrow beam of light is shined through the pupil;
Grade 2: The iris appears to be slightly thinned, and the pigmentation is slightly patchy when illuminated with a broad beam of light, and a small amount light is transmitted through the iris, typically in the mid-peripheral region of some areas, when a narrow beam of light is shined through the pupil;
Grade 3: The pigmentation of the iris is noticeably patchy, and the pupil has started losing its contour when illuminated with a broad beam of light, and diffuse light is transmitted across the entire iris when a narrow beam of light is shined through the pupil; and
Grade 4: The iris has a very patchy pigmentation pattern, and the pupil has lost its contour when illuminated with a broad beam of light, and an equal amount of light is emitted through the iris 360° when a narrow beam of light is shined through the pupil.

### Application of iris transillumination defect grading system

**Figure 2:**
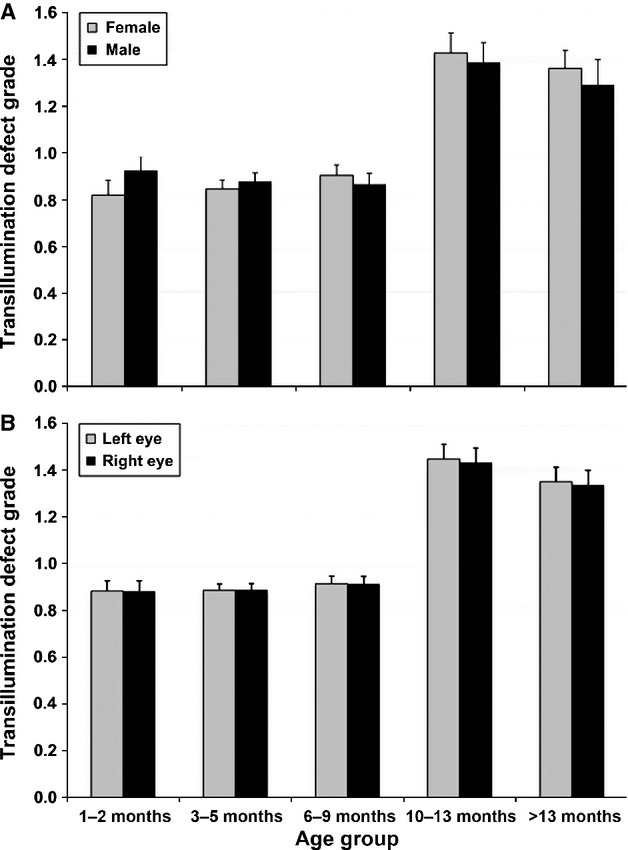
Iris transillumination defect segregated by age group and gender (A) or side (B). Within an age group, there were no statistical differences in TID grade. Therefore, data generated from females and males could be pooled within an age group, as could data from both left and right eyes.

After optimizing the TID grading scale, we applied it to mice from all BXD strains in each age group, as well as parents and F1 crosses. To determine whether data could be pooled across genders and within a mouse, separate analyses were performed on mice from each age cohort. Within each group, no significant differences were observed between females and males (Figure 2A, P > 0.05). Similarly, within each cohort, no significant difference was observed between right and left eyes (Figure 2B, P > 0.05). Moreover, the grade of TIDs from males/females and left/right eyes showed a high degree of correlation (*r* > 0.95, data not shown). Because our data lacked both gender and sidedness biases, we pooled all data for each BXD strain into a single data set that was stratified only by age.

**Figure 3:**
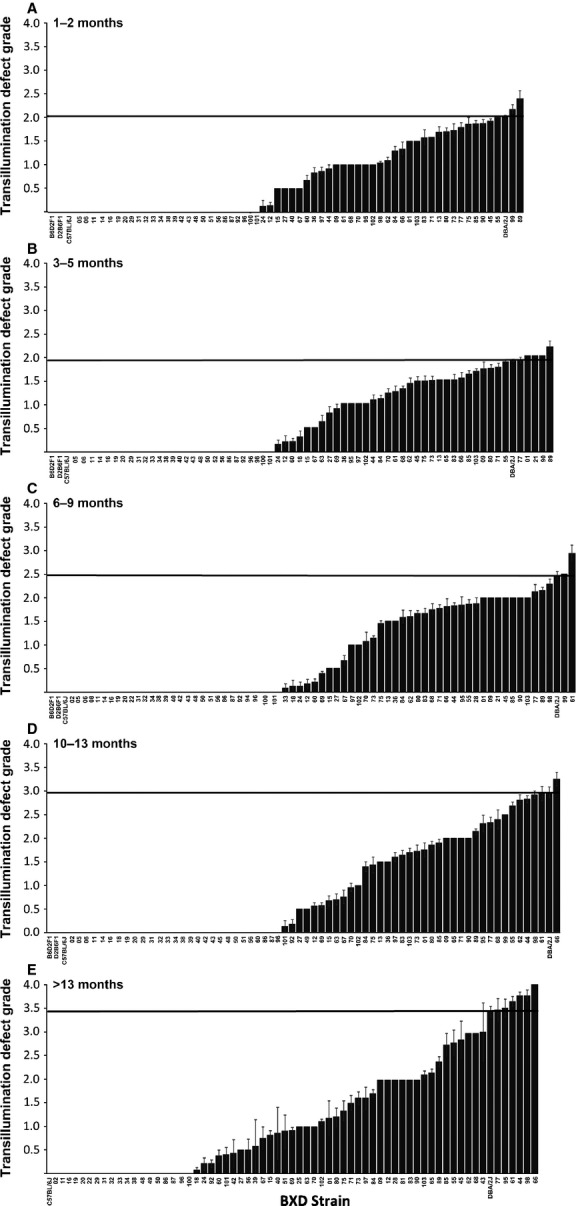
Iris transillumination defect of each BXD strain segregated by age group. (A) = mice at 1–2 months; (B) = mice at 3–5 months; (C) = mice at 6–9 months; (D)=mice at 10–13 months; and (E) = mice at >13 months. The horizontal line in each graph indicates the TID grade for the D2 parent.

The average grade of TIDs of DBA/2J and C57BL/6J parents was significantly different at all ages (Figure 3, P < 0.05). C57BL/6J parents from all cohorts presented a grade of 0. In contrast, DBA/2J parents had defects that averaged a grade of 2.02 ± 0.21 as early as 1–2 months of age and increased to a maximum of 3.45 ± 0.13 (>13 month). Both F1 hybrids (i.e., B6D2F1 and D2B6F1) demonstrated 0 grade at all ages (Figure 3).

Across the BXD strains, the degree of TID varied considerably and in many strains the defect increased with age. Twenty-two strains of BXD mice show no sign of TIDs at any age. In contrast, 55 strains had some degree of iris TID. Of these, the TID grade varied by no more than 1.0 between groups in 41 strains, while the TID grade varied by more than 1.0 between age groups in the remaining 14 strains. BXD66 mice >13 months old had the highest level of TID with value of grade 4.00 ± 0.00 (mean±SE) (Figure 3). Complete raw data is available at http://www.genenetwork.org/webqtl/main.py. The following are brief step-by-step instructions to be followed to access the TID data: Species>Mouse; Group>BXD; Type>Phenotypes; Data Set>BXD Published Phenotypes; Get any>Iris.

**Figure 4:**
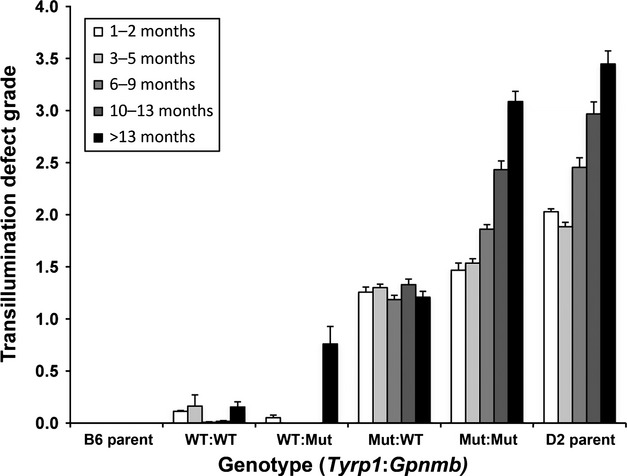
Iris transillumination defect for each age group segregated by genotype of *Tyrp1* and *Gpnmb*.

When the TID was stratified by both age and the genotypes at *Tyrp1* and *Gpnmb*, several findings became clear (Figure 4). Specifically, BXD strains carrying wild-type versions of both *Tyrp1* and *Gpnmb* had minimal TIDs, although they were slightly greater than the B6 parent at all ages. This lends support for the theory that the genetic D2 background harbors susceptibility genes (Anderson et al., 2001, 2008). BXD strains carrying only the mutant form of *Gpnmb* had no TID until they were aged over 13 months, while those carrying only the mutant form of *Tyrp1* had a significant TID that was unchanged with age. The presence of the mutation in *Gpnmb*, in addition to that in *Tyrp1*, significantly increased the TID in mice of all age groups (P < 0.01, anova followed by Scheffe's Post Hoc test). Finally, BXD strains carrying mutant versions of both *Tyrp1* and *Gpnmb* had significant TIDs at all ages that were less than the D2 parent, which lends support for the theory that the B6 genetic background harbors protective genes (Anderson et al., 2001).

### Heritability, QTL mapping of the TID, and identification of pigmentation gene modifiers of the TID

**Figure 5:**
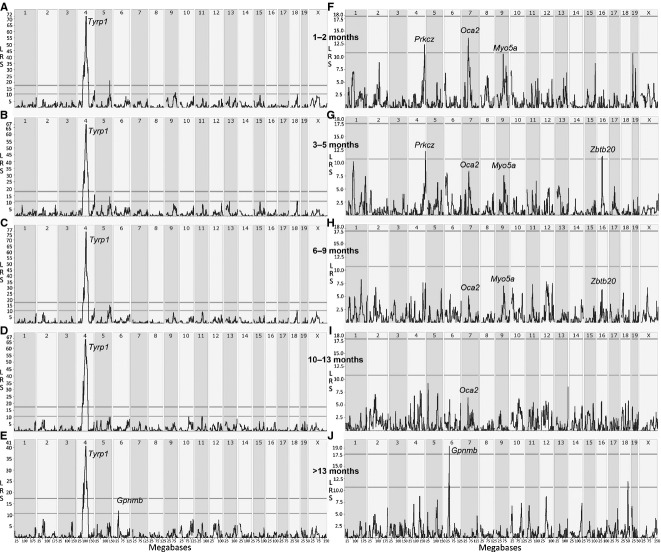
Genome-wide linkage simple (left column) and composite (right column) interval maps of grade of iris transillumination defects (TIDs). (A and F), (B and G), (C and H), (D and I), and (E and J) show linkage maps generated from mice aged 1–2, 3–5, 6–9, 10–13, and>13 months, respectively. The horizontal lines indicate levels of statistical significance with the upper line indicating statistical significance and the lower line indicating a statistically suggestive threshold. All simple interval maps have a significant QTL on chromosome 4 centered around 75–81 Mb, which is attributable to *Tyrp1*. In contrast, only the simple interval map from mice aged >13 months has a QTL peak, albeit suggestive, on chromosome 6 centered around 46–49 Mb, which is attributable to *Gpnmb*. The composite interval maps show QTLs that contribute to the TID phenotype. Of those, only 4 harbor pigmentation genes – *Oca2*, *Myo5a*, *Prkcz,* and *Zbtb20*.

Heritability of the TID ranged between 77.8–92.6% across the cohorts. Five individual simple interval maps, one for each age cohort, were generated to determine the genomic regions to which the TID maps (Figure 5). All age groups show a highly significant QTL on chromosome 4 centered between 75–81 Mb (Figure 5A–E). The likelihood ratio statistic (LRS) scores of these maps range from a high of 76.9 at 6–9 months of age to a low of 38.2 in mice aged >13 months. Located in the center of the peak QTL is *Tyrp1* (mapping at Chr 4 at 80.492 Mb), a gene known to be causative for the TID in D2 mice (Anderson et al., 2001; Lu et al., 2011a). Specific details on TID-modifier genes including chromosomal position and flanking markers are presented in Appendix S1. In the oldest cohort, a suggestive QTL (LRS = 12.6) on Chr 6 centered at 46–49 Mb was found. Located in the center of the peak QTL is *Gpnmb* (mapping at Chr 6 at 49.006 Mb), the second gene known to be causative for the TID in D2 mice (Anderson et al., 2001; Lu et al., 2011b). Interestingly, the *Gpnmb* peak was not present in the maps generated from the younger age groups. Simple interval maps generated from TID data from young mice showed a significant QTL peak on distal Chr 5 (Figure 5A,B). Because this LRS peak was no longer present in composite interval maps in which we controlled for the influence of the large QTL attributed to *Tyrp1*, it is not a valid independent QTL and was pursued no further. Very likely, the apparent QTL on Chr 5 was due to the population substructure in BXD strains and the associated observation that markers on distal Chr 5 are genetically linked to markers for *Tyrp1*.

Composite interval maps for all five age cohorts revealed several secondary peaks that were masked by the presence of the large influence of the *Tyrp1* peak (Figure 5F–J). Specifically, in the oldest cohort, the peak on Chr 6 attributable to *Gpnmb* at 48–50 Mb became statistically significant (LRS = 19.2, Figure 5J). In mice younger than 13 months, this peak on Chr 6 had LRS scores of 6 or less. Other secondary peaks that had fulfilled our criteria for a QTL were located on the following: Chr 4 (at ∼150 Mb) in mice aged 1–2 months (Figure 5F) and 3–5 months (Figure 5G); Chr7 (at ∼60–65 Mb) in mice aged 1–2 (Figure 5F), 3–5 (Figure 5G) and 6–9 (Figure 5H); Chr9 (at ∼70–75 Mb) in mice aged 1–2 (Figure 5F), 3–5 (Figure 5G) and 6–9 months (Figure 5H); Chr16 (at ∼45 Mb) in mice aged 3–5 months (Figure 5G) and 6–9 months (Figure 5H). In addition, there were as many as 36 other secondary QTLs (Figure 5F–J) that were screened further for candidate genes. To determine whether any of the secondary QTLs harbored pigmentation genes, we conducted an exhaustive search on the MGI phenotype database using ‘iris’ and ‘pigmentation’ as the search terms, which provided an impressive list of 1155 gene candidates. We determined whether any of those genes were located within the aforementioned shortlisted secondary QTL peaks in composite mapping (Figure 5F–J) and the 36 other secondary QTLs. Any gene candidate that was common in both lists and fulfilled our robust inclusion criteria were chosen for further analysis. Other than the known genes, *Tyrp1* and *Gpnmb*, we found only four other plausible candidate pigmentation genes—*Oca*, Myo5a, *Prkcz,* and *Zbtb20*—within the secondary QTL peaks, which fulfilled our inclusion criteria. Other pigmentation or iris disease-related genes that seemed compelling but did not fulfill our inclusion criteria are presented in Appendix S8. *Oca2* maps at Chr7 at 63.791 Mb, while *Myo5a* maps at Chr9 at 75.065 Mb. One of the critical parameters for evaluation was the presence of SNPs/Indels as shown in Appendices S1–S5. As per our previously published results, there were two known missense exonic SNPs in *Tyrp1* and an additional pair of synonymous exonic SNPs along with 42 SNPs at non-splice sites (Lu et al., 2011a). With these newer studies, we could embellish our data with an additional non-synonymous exonic SNP, two 3′UTR SNPs, 46 SNPs at non-splice sites, and three deletions (Appendix S2). For *Gpnmb*, we could gather more insight than our previously published data (Lu et al., 2011b) and found a total of 54 SNPs with a non-synonymous SNP on exon 2, a stop codon in exon 4, and three synonymous SNPs in exon 3, 4,,49 SNPs in non-splice sites, and two deletions (Appendix S3). For *Oca2*, there were a total of 970 SNPs, with three SNPs in exon 1, five each 3′UTR SNPs, a pair of non-synonymous SNPs in exons 23 and 24, and 25 Indels (Appendix S4). In case of *Myo5a*, there were 25 SNPs at non-splice sites and 35 Indels (Appendix S5). For *Prkcz*, there was 1 non-synonymous SNP in exon 7, 1 3′UTR SNP, and 9 SNPs at non-splice sites (Appendix S6). In case of *Zbtb20*, we found a total of 84 SNPs at non-splice sites, along with 3 non-synonymous SNPs in exon 8, with 2 insertions and 2 deletions (Appendix S7).

The six candidate genes that met our inclusion criteria were subjected to general linear modeling to evaluate the contribution of each gene individually as well as any possible interactions between the pigmentation genes. These results demonstrate that at different time points during the progression of the TID, all six genes contribute to the phenotype (Table 1). In some age groups, the genes contribute individually, while in others, they interact with one or two additional genes. We used up to a six-way analysis to check for the best possible combination of genes in our interaction model. We found that the lowest error values and highest per cent observed total variances were present using three-way analysis, and these values remained unchanged up to six-way analysis. Hence, we chose a 3-way model for our interaction studies. Using our ordinary least squares (OLS) three-way interaction model, we found that the predictors, that is, *Tyrp1* (T), *Gpnmb* (G), *Myo5a* (M), *Oca2* (O), *Prkcz* (P), and *Zbtb20* (Z), were highly significant in all age cohorts except the oldest one, that is, 13+ months of mice, where only G and T were found highly significant. With regard to the genetic interactions, we found significant cross-talk between the predictors at all age groups. The most common of those interactions involved *Tyrp1*, *Gpnmb,* or *Oca2*. The extent of genetic interactions was lowest in age cohort 13+ months. At this age cohort, none of the predictors other than *Tyrp1* and *Gpnmb* were highly significant. The detailed analyses are presented in Table 1. We also validated our results using sas statistical software, which showed identical results (data not shown).

**Table 1:** Statistical evaluation and resultant mathematical model for six causal genes

Age (month)	Additive Predictors	Interactions	Total explained variance (%)
1–2	**T, G, O, M, P, Z**	T*G, T*O, T*G*O, T*M, G*M, T*O*M, G*O*M, T*P, G*P, O*P, G*O*P, T*M*P, T*O*Z, O*P*Z, M*P*Z, O*M, M*Z, P*Z, T*P*Z	84.7
3–5	**T, G, O, M, P, Z**	T*G, T*O, G*O, T*G*O, T*M, G*M, T*G*M, O*M, T*O*M, G*O*M, T*P, G*P, O*P, T*O*P, T*M*P, T*Z, T*O*Z, G*M*Z, T*P*Z, O*P*Z, P*Z	81.4
6–9	**T, G, O, M, P, Z**	T*G, T*O, G*O, T*G*O, T*M, G*M, T*G*M, O*M, T*O*M, G*O*M, G*P, O*P, T*O*P, M*P, T*M*P, T*Z, T*O*Z, G*M*Z, P*Z, T*P*Z, O*Z, M*Z	79.7
10–13	**T, G, O, M, P, Z**	T*G, T*O, G*O, T*G*O, T*M, G*M, T*O*M, T*P, G*P, T*G*P, O*P, G*O*P, T*Z, G*Z, M*Z, G*M*Z, P*Z, T*P*Z, T*O*P, M*P	88.0
13+	**T, G**	M*Z, G*M*Z, P*Z, T*P*Z, T*G*P, O*P, G*O*P, T*Z	70.5

T, *Tyrp1*; G, *Gpnmb*; O, *Oca2*; M, *Myo5a*; P, *Prkcz*; Z, *Zbtb20*.

Significance levels of P ≤ 0.05 are indicated by the larger bold font, while significance levels of 0.05 ≤ P ≤ 0.1 are indicated by the normal font.

Mathematical Model: Variance in the iris transillumination defect can be computed using the formula: V = (P1+P2+P3+P4+P5+P6) + (P1*P2) + (P1*P2*P3) + (P1*P2*P3*P4) + (P1*P2*P3*P4*P5) + (P1*P2*P3*P4*P5*P6) + E.

V, Variance; P, Predictors; E, Error term.

## Discussion

To gain an understanding of polygenetic nature of a quantitative phenotype, the contributions of multiple genes must be simultaneously evaluated. This can be an arduous task under the best circumstances and nearly impossible when using a genetically diverse reference panel. In this study, we demonstrate for the first time the simultaneous contributions of multiple genes to the TID phenotype. Using our large repository of phenotypic and genetic data along with the bioinformatics tools available on GeneNetwork, we have generated a mathematical model that accounts for 71–83% of the genetic factors that modulate the TID.

Over a decade ago, the John laboratory determined that mutations in two interacting genes—*Tyrp1* and *Gpnmb*—cause severe iris atrophy and pigmentary dispersion, respectively, in D2 mice (Anderson et al., 2001; Chang et al., 1999; John et al., 1998). Other groups have documented that the expression of this phenotype is variable (Trantow et al., 2011). We hypothesized that other pigmentation genes modulate the primary causal genes to generate this variability. In this investigation, we have used a systems genetics approach to identify the genetic modifiers of TID with an emphasis on known pigmentation genes.

Our study has demonstrated that from birth, the TID maps to *Tyrp1*, indicating that this gene plays a major role in the generation of this phenotype. Our data also demonstrate that the phenotype is not progressive in BXD strains that harbor only the mutant form of *Tyrp1*, suggesting that the ISA is a developmental rather than a degenerative phenotype. The addition of the mutant form of *Gpnmb*, which increases the amount of pigment that sloughs from the iris, transforms the TID into a phenotype that progressively worsens with age. Interestingly, the presence of only the *Gpnmb* mutation results in the absence of a TID until mice are >13 months of age. The presence of both mutations results in an apparent interaction of these genes, although the effect of *Gpnmb* by mapping studies is not overtly evident until >13 months. These puzzling data lead us to speculate that other pigmentation genes also influenced the full manifestation of the TID. To evaluate this hypothesis, we systematically evaluated each LRS peak in the composite interval maps to determine whether any of them contained genes known to be involved in pigmentation using the stringent criteria outlined in the methods section. Further, on conducting an exhaustive search on the MGI phenotype database using ‘iris’ and ‘pigmentation’ as the search terms, we arrived at an impressive list of 1155 gene candidates. After comparing the aforementioned shortlisted secondary QTL peaks from composite mapping (Figure 5F–J) with the MGI database list of 1155 genes, we determined that only four additional pigmentation genes—*Oca2*, *Myo5a*, *Prkcz* and *Zbtb20* — fulfilled our criteria.

The literature contains reports on the association of genes such as *Lyst* (Trantow et al., 2009) and *Rab38* (Brooks et al., 2007) to iris-related phenotypes. We did not include these genes in our model of TID because they did not fulfill our inclusion criteria. Specifically *Lyst*, also known as lysosomal trafficking regulator, is located on Chr 13 at 13.870 Mb. As it can be seen in Figure 5F–J, there are no QTLs present at this location; hence, we did not explore it further. A similar strategy was followed for the evaluation of *Rab38*, a member of *Ras* oncogene family. Groups have also shown that mutations in *Dct* (dopachrome tautomerase) affect eumelanin/pheomelanin synthesis, but do not affect intracellular trafficking of the mutant protein, leading to a mild iris phenotype (Costin et al., 2005). Despite this biological significance, *Dct* did not fulfill our inclusion criteria. We were interested in genes that were common to both the MGI phenotype database list and our list obtained from the QTL studies in GeneNetwork. Other genes associated with pigmentation or iris-related phenotypes (Frudakis et al., 2003; Sturm et al., 2001; Tully, 2007) were also screened for their presence in our QTL maps; a summary table highlighting the genes that were excluded from the analysis is presented in Appendix S8. As can be in the Appendix, none of these genes qualified for inclusion in our current study. We also evaluated all other QTL peaks in Figure 5 for known pigmentation genes or genes causing iris phenotypes. After carefully evaluating all genes under each LRS peak, we failed to find any additional candidates that could be included in our model. Additional analyses to evaluate these candidates in TID are beyond the scope of the current work and are the subject of a future investigation to understand further genetic accomplices of TID.

All six candidate genes that we identified in this investigation—*Tyrp1*, *Gpnmb*, *Oca2*, *Myo5a*, *Prkcz,* and *Zbtb20*—are expressed in the iris, thereby lending strong support to the interaction of the genes and their respective gene products. As stated previously, *Tyrp1* is expressed in the iris stroma, while *Gpnmb* is expressed by the iris pigment epithelium (Chang et al., 1999; Howell et al., 2007). With age, mutations in these genes synergize, leading to iris disease in D2 mice that can be genetically separated into two distinct traits—IPD and ISA (Chang et al., 1999; Howell et al., 2007). Genetics Home Reference (NIH) searches reveal that both *Myo5a* and *Oca2* are abundant in melanocytes, which are present in both the neural crest cell-derived iris stroma and pigment epithelium. MYO5A functions as a motor protein and is critical for melanogenesis, while OCA2 provides instructions for making P protein, which plays a role in maintaining the acidity of melanosomes within melanocytes. In addition, our previous study has demonstrated that *Myo5a* regulates *Tyrp1* gene expression (Lu et al., 2011a). Our current mapping studies, combined with interactions from mathematical modeling and a strong biological synergy, provide a strong biological basis for interactions at the genetic and gene product levels. The role of *Prkcz* in melanocyte formation is well documented, and it has been reported that the primary signaling pathway for lysophosphatidylcholine-based dendrite formation in human melanocytes involves the activation of PRKCZ (Scott et al., 2007). ZBTB20 plays a role in metal ion binding to melanosomes especially that of zinc. The role of ions such as zinc in the eye has been studied in the past, and it is evident that all the melanocytes are high in zinc content. Also the presence of metals in the catalytic centers of melanosomal enzymes is well known. Thus, inclusion of *Zbtb20* in our model is well justified (Borovansky and Riley, 2011).

Our mathematical model accounts for 71–88% of the explained variance of the TID phenotype across the age bins that we investigated, leaving approximately 10% of the genetic contribution toward the TID unaccounted for by these six genes. We used up to a six-way analysis to check for the best possible combination of genes in our mathematical model. Lowest error values and highest per cent observed total variances were seen using a three-way analysis; hence, we chose a 3-way model for our interaction studies. The results were in agreement with the mapping studies as seen in the Figure 5, wherein *Gpnmb* and *Tyrp1* are the only genes having a high level of significance at the oldest cohort and other predictors contributing at one or more age cohorts. We also validated our results using sas statistical software, which provided identical results (data not shown). It is our goal to explore the genetic modulation of the TID phenotype in greater depth using an innovative Quantitative Trait SNPs approach, which may shed light on the remaining genes associated with the TID phenotype. Similar methods can be used to determine the genetic complexities of other phenotypes including polygenetic diseases such as glaucoma, age-related macular degeneration, Alzheimer's disease, and diabetes.

## Methods

### BXD RI mice

Mice were handled in a manner consistent with the Guide for the Care and Use of Laboratory Animals (Institute of Laboratory Animal Resources, the Public Health Service Policy on Humane Care and Use of Laboratory Animals), and all studies were approved the Animal Care and Use review board of the University of Tennessee Health Science Center, an AAALAC-accredited facility. Mice were maintained at a temperature of 20–24°C on a 12:12 light/dark cycle with 35–40% humidity in a specific pathogen-free (SPF) environment at the University of Tennessee Health Science Center Animal Facility. Animals were weaned at 25 days of age, housed in same-sex cages (2–5 mice per cage), and were fed 5% fat Agway Prolab 3000 rat and mouse chow.

A total of 3564 mice were used in this study: 3280 mice from 73 BXD RI strains; 127 D2 mice; 83 B6 mice; 51 B6D2F1 mice; and 23 D2B6F1 mice. The gender distribution was relatively equal with 1965 females and 1599 males being included. Because the transillumination defect of D2 mice can be progressive (John et al., 1998; Libby et al., 2005), mice were segregated by age and grouped by the categories of 1–2, 3–5, 6–9, 10–13, and >13 months).

### BXD Genotypes database and mapping algorithm in GeneNetwork

Readers are directed to the works of Shifmann et al. (Shifman et al., 2006) and Williams et al. (Williams et al., 2001) for a detailed description of the genotyping methodology and the algorithms of GeneNetwork. Our research groups and collaborators have extensively published genetic modulation studies using GeneNetwork (Geisert et al., 2009; Jablonski et al., 2011; Lu et al., 2011a,b). Briefly, the BXD genotype file was created using high-density Affymetrix arrays, which exploit a set of 3795 markers typed across 88 extant and extinct BXD strains (BXD1 through BXD100). This genotype file includes all markers, both SNPs and microsatellites, with unique strain distribution patterns (SDPs), as well as pairs of markers for those SDPs represented by two or more markers. The latest smoothed BXD genotype data file can be downloaded by ftp from GeneNetwork at the URL http://www.genenetwork.org/genotypes/BXD.geno.

In collaboration with members of the Complex Trait Consortium (Drs. Richard Mott, Jonathan Flint, and colleagues), we have helped genotype a total of 480 strains using a panel of 13,377 SNPs. These SNPs have been combined with our previous microsatellite genotypes to produce consensus maps for the expanded set of BXD using the latest mouse genome assembly as a reference frame for marker order (Mouse Build 36 - UCSC mm8). The order of markers given in the BXD genotype file is essentially the same as that given in Build 36. (Files were updated from mm6 to mm8 in January 2007.). Post-July 2007, we have been using NCBI37/mm9 assembly obtained from NCBI and the Mouse Genome Sequencing Consortium.

A total of 88 strains were genotyped using the full set of SNPs, and 7482 of these were informative. Informative in this sense simply means that the C57BL/6J and DBA/2J parental strains have different alleles. To reduce false-positive errors when mapping using this ultradense map, we have eliminated most single genotypes that generate double-recombinant haplotypes that are most commonly produced by typing errors (‘smoothed’ genotypes). For this reason, the genotypes used in the GeneNetwork differ from those downloaded directly from Richard Mott's web site at the Wellcome Trust, Oxford. Due to the very high density of markers, the mapping algorithm used to map BXD data sets has been modified and is a mixture of simple marker regression, linear interpolation, and standard Haley–Knott interval mapping. When two adjacent markers have identical SDPs, they will have identical linkage statistics, as will the entire interval between these two markers (assuming complete and error-free haplotype data for all strains). Readers are also directed to the following links for further support with GeneNetwork: http://www.genenetwork.org/reference.html; http://www.genenetwork.org/links.html; http://www.genenetwork.org/tutorial/WebQTLTour/; and http://www.genenetwork.org/glossary.html. These links would provide a more comprehensive list of references (some with pdf links), tutorials, glossary of terms, and an exhaustive list of resources used for the functioning of GeneNetwork.

### Grading of iris transillumination defects in BXD RI mice

Previous studies have shown that the anterior segment disorder of DBA/2J mouse includes pigment dispersion and iris atrophy with associated synechiae (Anderson et al., 2008; John et al., 1998; Libby et al., 2005). Although some strains of BXD mice have varying degrees of pigment dispersion or/and iris atrophy, individually, each defect is difficult to quantify. We found by slit-lamp biomicroscope examination that some mice have TIDs without obvious pigment dispersion or iris atrophy, indicating that it is difficult to detect minor degrees of each defect individually. We have found that the collective TID is a sensitive phenotype and encompasses both the degree of pigment dispersion and iris atrophy. Moreover, it is relatively easy to divide this defect to several quantitative levels.

After lightly anesthetizing the mice with ketamine and xylazine, the iris phenotype of each mouse was assayed with a slit-lamp microscope (SL-D7; Topcon, Tokyo, Japan) and photodocumented with a digital camera (D100; Nikon, Tokyo, Japan). For assessment of TIDs, a small beam of light was shone directly through the slit of the dilated pupil of the mouse, and the iris was examined for the ability of reflected light to pass through depigmented areas of the iris. All photographs were taken with identical camera settings and were prepared with identical image software processing. After optimizing the TID grading scale, we applied it to mice from all BXD strains in each age group, as well as parents and F1 crosses. To determine whether data could be pooled across genders and within a mouse, separate analyses were performed on mice from each age cohort. We also stratified TID by both age and the genotypes at *Tyrp1* and *Gpnmb* and analyzed the data statistically using an anova with Scheffe's post hoc tests.

### Left and right side asymmetry and gender asymmetry

The grade of TID of left and right eyes of all mice was measured and pooled separately within each age group. A paired t-test was used to statistically compare the values of TID grades between eyes. Likewise, a paired t-test was used to compare the TID grades between females and males of each age group. All statistical evaluations were performed using datadesk 6.3 (Data Description, Inc., Ithaca, NY, USA).

### Heritability calculation and QTL mapping

Heritability of the TID phenotype was calculated using the formula: *h*_2_ = 0.5V_g_/(0.5V_g_ + V_e_) (Hegmann and Possidente, 1981) where h_2_ is the heritability, V_g_ is the genetic variance, and V_e_ is the environmental variance. The factor of 0.5 in this ratio was applied to adjust for the two-fold increase in additive genetic variance among inbred strains relative to outbred populations (Geisert et al., 2009; Lu et al., 2011a,b).

Grades of TIDs data from BXD RI strains were used to map potential QTLs. QTL maps were generated using GeneNetwork (http://www.genenetwork.org/) a set of 3795 markers. Linkage is reported with genome-wide significance levels based on 2000 permutation tests. Two types of QTL mapping analyses–simple mapping using the Haley–Knott regression equation, and composite interval mapping–were utilized in this study. Simple interval mapping was performed to illustrate the significance of any QTLs that regulate the TID. As a secondary analysis, composite interval mapping which controlled for the influence of *Tyrp*1 was also performed with the goal of identifying any secondary QTLs that may have been masked by the major QTL on Chr 4. Each of these analyses produced a LRS score, which is a chi-square statistic that provides a measure of the linkage between variation in the phenotype and genetic differences at a specific genetic locus. Logarithm of odds values can be obtained simply by dividing the LRS by 4.61. Further, we conducted an exhaustive search on the MGI phenotype database using ‘iris’ and ‘pigmentation’ as the search terms to shortlist known pigmentation gene candidates playing a role in iris-related diseases. We examined all the genes obtained within the secondary QTL peaks in composite mapping (Figure 5F–J). Any gene candidate that was common in both lists was chosen for further screening using the inclusion criteria mentioned below.

### Evaluation of candidate pigmentation genes as potential modifiers of *Tyrp1* and *Gpnmb*

To determine whether any genes known to be involved in pigmentation may modify the TID along with *Tyrp1* and *Gpnmb* we used the following criteria:

1. The known pigmentation gene was located ± 5 Mb of the QTL peaks in any composite interval map;
2. The LRS peak containing the pigmentation gene was at suggestive level or higher in at least one age cohort and measured above the background in at least one additional age cohort;
3. The gene had sequence variants (SNPs and/or indels) between the parental strains as determined via the variant browser link on GeneNetwork;
4. The gene had expression differences between the parental strains as determined via the RNA-Seq link on GeneNetwork; and
5. The mean expression level of the gene across the BXD strains was >7, in the age group in which its LRS score was at or above the suggestive levels in GeneNetwork.

### Mathematical modeling

A general linear model using OLS with up to six-way interactions was used to evaluate the contributions of individual genes as well as potential interactions between the various genes. The identical analysis was run on all possible combinations of the six pigmentation genes. The analysis that produced the highest explained variance was used in our model. The explained variance was calculated as (total variance-error term)/total variance*100. For all age groups, inclusion of all six pigmentation genes provided highest explained variance (Table 1). Only those pigmentation genes that fulfilled the above criteria were included in our statistical evaluation and mathematical modeling. In this analysis, we determined the contribution of each candidate gene individually as well its interactions with other pigmentation genes for each age group. Iris transillumination defect grades were scored as a function of the age, strain, and genotypes for each gene candidate, and the data were analyzed using general linear models (GLM) with genotypes as fixed factors using DataDesk 6.3 statistical software (Ithaca, NY). Different permutations and combinations of the pigmentation genes (i.e., *Gpnmb*, *Tyrp1*, *Oca2*, *Myo5a*, *Prkcz* and *Zbtb20*) were included into the model, and the contribution of individual genes and potential interactions were studied. A general linear model using OLS with up to six-way interactions was used to evaluate the contributions of individual genes as well as potential interactions between the various genes. General linear models are a flexible generalization of ordinary linear regression that allows for response variables that have other than a normal distribution. The GLM generalizes linear regression by allowing the linear allows the magnitude of the variance of each measurement to be a function of its predicted value. The model was devised with the following formula, wherein the variance in the TID was computed as follows: V = (P1+P2+P3+P4+P5+P6) + (P1*P2) + (P1*P2*P3) + (P1*P2*P3*P4) + (P1*P2*P3*P4*P5) + (P1*P2*P3*P4* P5*P6) + E; where, V = Variance; P = Predictors (the six causal genes); and E = Error term. Significance levels of P ≤ 0.05 were considered highly significant, while significance levels of 0.05 ≤ P ≤ 0.1 were considered marginally significant. The predictors yielding the highest variance, and the lowest error terms were selected for modeling. We also validated our results from DataDesk using sas statistical software with the option, anova for unequal cell size for each outcome, and Type III sum of squares.

## Supporting information

**Appendix S1.** Summary of candidate genes.

**Appendix S2.** List of SNPs within *Tyrp1*.

**Appendix S3.** List of SNPs within *Gpnmb*.

**Appendix S4.** List of SNPs within *Oca2*.

**Appendix S5.** List of SNPs within *Myo5a*.

**Appendix S6.** List of SNPs within *Prkcz*.

**Appendix S7.** List of SNPs within *Zbtb20*.

**Appendix S8.** Screening of known pigmentation and iris phenotype related genes.

## References

[b1] Anderson MG, Smith RS, Hawes NL, Zabeleta A, Chang B, Wiggs JL, John SW (2001). Mutations in genes encoding melanosomal proteins cause pigmentary glaucoma in DBA/2J mice. Nat. Genet.

[b2] Anderson M, Hawes N, Trantow C, Chang B, John S (2008). Iris phenotypes and pigment dispersion caused by genes influencing pigmentation. Pigment Cell Melanoma Res.

[b3] Ball SF (2004). Pigmentary Glaucoma.

[b4] Bennett DC, Lamoreux ML (2003). The color loci of mice –a genetic century. Pigment Cell Res.

[b5] Borovansky J, Riley PA (2011). Melanins and Melanosomes: Biosynthesis, Biogenesis, Physiological and Pathological Functions.

[b6] Brooks BP, Larson DM, Chan CC (2007). Analysis of ocular hypopigmentation in Rab38cht/cht mice. Invest. Ophthalmol. Vis. Sci.

[b7] Chang B, Smith RS, Hawes NL, Anderson MG, Zabaleta A, Savinova O, Roderick TH, Heckenlively JR, Davisson MT, John SW (1999). Interacting loci cause severe iris atrophy and glaucoma in DBA/2J mice. Nat. Genet.

[b8] Costin GE, Valencia JC, Wakamatsu K (2005). Mutations in dopachrome tautomerase (Dct) affect eumelanin/pheomelanin synthesis, but do not affect intracellular trafficking of the mutant protein. Biochem. J.

[b9] Fine BS, Yanoff M (1979). Ocular Histology.

[b10] Frudakis T, Thomas M, Gaskin Z, Venkateswarlu K, Chandra KS, Ginjupalli S, Gunturi S, Natrajan S, Ponnuswamy VK, Ponnuswamy KN (2003). Sequences associated with human iris pigmentation. Genetics.

[b11] Geisert EE, Lu L, Freeman-Anderson NE, Templeton JP, Nassr M, Wang X, Gu W, Jiao Y, Williams RW (2009). Gene expression in the mouse eye: an online resource for genetics using 103 strains of mice. Mol. Vis.

[b12] Hegmann JP, Possidente B (1981). Estimating genetic correlations from inbred strains. Behav. Genet.

[b13] Hirobe T, Abe H (2007). Changes of melanosome morphology associated with the differentiation of epidermal melanocytes in slaty mice. Anat. Rec. (Hoboken).

[b14] Hong L, Simon JD, Sarna T (2006). Melanin structure and the potential functions of uveal melanosomes. Pigment Cell Res.

[b15] Howell GR, Libby RT, Marchant JK, Wilson LA, Cosma IM, Smith RS, Anderson MG, John SWM (2007). Absence of glaucoma in DBA/2J mice homozygous for wild-type versions of Gpnmb and Tyrp1. BMC Genet.

[b16] Hu DN (2005). Photobiology of ocular melanocytes and melanoma. Photochem. Photobiol.

[b17] Iwata F, Reed GF, Caruso RC, Kuehl EM, Gahl WA, Kaiser-Kupfer MI (2000). Correlation of visual acuity and ocular pigmentation with the 16-bp duplication in the HPS-1 gene of Hermansky-Pudlak syndrome, a form of albinism. Ophthalmology.

[b18] Jablonski MM, Freeman NE, Orr WE, Templeton JP, Lu L, Williams RW, Geisert EE (2011). Genetic pathways regulating glutamate levels in retinal Muller cells. Neurochem. Res.

[b19] John SW, Smith RS, Savinova OV, Hawes NL, Chang B, Turnbull D, Davisson M, Roderick TH, Heckenlively JR (1998). Essential iris atrophy, pigment dispersion, and glaucoma in DBA/2J mice. Invest. Ophthalmol. Vis. Sci.

[b20] Kobayashi T, Imokawa G, Bennett DC, Hearing VJ (1998). Tyrosinase stabilization by Tyrp1 (the brown locus protein). J. Biol. Chem.

[b21] Li L, Hu DN, Zhao H, McCormick SA, Nordlund JJ, Boissy RE (2006). Uveal melanocytes do not respond to or express receptors for alpha-melanocyte-stimulating hormone. Invest. Ophthalmol. Vis. Sci.

[b22] Libby RT, Anderson MG, Pang I-H (2005). Inherited glaucoma in DBA/2J mice: pertinent disease features for studying the neurodegeneration. Vis. Neurosci.

[b23] Lin JY, Fisher DE (2007). Melanocyte biology and skin pigmentation. Nature.

[b24] Liu Y, Hong L, Wakamatsu K, Ito S, Adhyaru BB, Cheng CY, Bowers CR, Simon JD (2005). Comparisons of the structural and chemical properties of melanosomes isolated from retinal pigment epithelium, iris and choroid of newborn and mature bovine eyes. Photochem. Photobiol.

[b25] Lu H, Li L, Watson E, Williams R, Geisert E, Jablonski M, Lu L (2011a). Complex interactions of Tyrp1 in the eye. Mol. Vis.

[b26] Lu H, Wang X, Pullen M (2011b). Genetic dissection of the Gpnmb network in the eye. Invest. Ophthalmol. Vis. Sci.

[b27] Lynch S, Yanagi G, Delbbono E, Wiggs JL (2002). DNA sequence variants in the tyrosinase-related protein 1 (TYRP1) gene are not associated with human pigmentary glaucoma. Mol. Vis.

[b28] Matsunaga J, Riley PA, Soloano F, Hearing VJ (2002). Catecholamine Research: From Molecular Insights to Clinical Medicine. Advances in Behavioral Biology.

[b29] Moyer FH (1966). Genetic variations in the fine structure and ontogeny of mouse melanin granules. Am. Zool.

[b30] Paigen K (2003). One hundred years of mouse genetics: an intellectual history. I. The classical period. Genetics.

[b31] Ritch R, Shields MB, Krupin T (1996). The Glaucomas.

[b32] Rodrigues MM, Hackett J, Donohoo P (1982). Ocular Anatomy, Embryology, and Teratology.

[b33] Scott GA, Arioka M, Jacobs SE (2007). Lysophosphatidylcholine mediates melanocyte dendricity through PKCzeta activation. J. Invest. Dermatol.

[b34] Shifman S, Bell TJ, Copley RR, Taylor MS, Williams RW, Mott R, Flint J (2006). A high-resolution single nucleotide polymorphism genetic map of the mouse genome. PLoS Biol.

[b35] Shuba L, Nicolela MT, Rafuse PE (2007). Correlation of capsular pseudoexfoliation material and iridocorneal angle pigment with the severity of pseudoexfoliation glaucoma. J. Glaucoma.

[b36] Smith-Thomas LC, Moustafa M, Dawson RA, Wagner M, Balafa C, Haycock JW, Krauss AH, Woodward DF, MacNeil S (2001). Cellular and hormonal regulation of pigmentation in human ocular melanocytes. Pigment Cell Res.

[b37] Solano F, Hearing VJ, Garcia-Borron JC (2000). Neurotoxicity due to o-quinones: neuromelanin formation and possible mechanisms for o-quinone detoxification. Neurotox. Res.

[b38] Sowka J (2004). Pigment dispersion syndrome and pigmentary glaucoma. Optometry.

[b39] Sturm RA, Teasdale RD, Box NF (2001). Human pigmentation genes: identification, structure and consequences of polymorphic variation. Gene.

[b40] Summers CG (1996). Vision in albinism. Trans. Am. Ophthalmol. Soc.

[b41] Tai T, Eisinger M, Ogata S, Lloyd KO (1983). Glycoproteins as differentiation markers in human malignant melanoma and melanocytes. Cancer Res.

[b42] Trantow C, Mao M, Petersen G, Alward E, Alward W, Fingert J, Anderson M (2009). Lyst mutation in mice recapitulates iris defects of human exfoliation syndrome. Invest. Ophthalmol. Vis. Sci.

[b43] Trantow C, Hedberg-Buenz A, Iwashita S, Moore S, Anderson M (2010). Elevated oxidative membrane damage associated with genetic modifiers of lyst-mutant phenotypes. PLoS Genet.

[b44] Trantow CM, Cuffy TL, Fingert JH, Kuehn MH, Anderson MG (2011). Microarray analysis of iris gene expression in mice with mutations influencing pigmentation. Invest. Ophthalmol. Vis. Sci.

[b45] Tully G (2007). Genotype versus phenotype: human pigmentation. Forensic Sci. Int. Genet.

[b46] Ward WC, Simon JD (2007). The differing embryonic origins of retinal and uveal (iris / ciliary body and choroid) melanosomes are mirrored by their phospholipid composition. Pigment Cell Res.

[b47] Williams RW, Gu J, Qi S, Lu L (2001). The genetic structure of recombinant inbred mice: high-resolution consensus maps for complex trait analysis. Genome Biol.

[b48] Winder A, Kobayashi T, Tsukamoto K, Urabe K, Aroca P, Kameyama K, Hearing VJ (1994). The tyrosinase gene family – interactions of melanogenic proteins to regulate melanogenesis. Cell. Mol. Biol. Res.

[b49] Wistow G, Bernstein SL, Ray S, Wyatt MK, Behal A, Touchman JW, Bouffard G, Smith D, Peterson K (2002). Expressed sequence tag analysis of adult human iris for the NEI Bank Project: steroid-response factors and similarities with retinal pigment epithelium. Mol. Vis.

[b50] Wong VW, Lam PT, Lai TY, Lam DS (2005). Black diaphragm aniridia intraocular lens for aniridia and albinism. Graefes Arch. Clin. Exp. Ophthalmol.

