# Supplementary material for: Genetic modulation of the iris transillumination defect: a systems genetics analysis using the expanded family of BXD glaucoma strains
Source: Pigment Cell Melanoma Res. 2013 Apr 13;26(4):487–98. doi: 10.1111/pcmr.12106 (PMC3752936; doi:10.1111/pcmr.12106)
Supplement: Supplementary file 1 [file pcmr0026-0487-SD1.pdf]

Appendix 1: Summary of candidate genes

Gene i.d.	Location	Max LRS value and location	Probe set i.d. and BLAT specificity	Markers flanking the gene	Mean expression level	SNPs and Indels
Tyrp1	Chr4: 80.492 Mb (80834123-80851719 bp) on the plus strand	76.91 (6-9 months, Figure 5C); Chr4: 78.698 Mb	1415862_at; 2.5	rs3658567-76.484 Mb, rs3708061-80.950 Mb	12.798	• 1 each non-synonymous SNPs on exons 2, 3 and 5<br>• 1 each synonymous SNP on exon 4 and exon 6<br>• 2 each 3'UTR SNPs<br>• 46 SNPs at non-splice sites<br>• 3 deletions
Gpnmb	Chr6: 49.00 Mb (49036518-49058182 bp) on the plus strand	20.0 (>13 months, Figure 5J); Chr6: 48.92 Mb	1448303_at; 10.5	rs13478744-48.680 Mb, rs4225888-49.078 Mb	11.189	• 1 non-synonymous SNP in exon 2<br>• 1 stop codon in exon 4<br>• 1 each synonymous SNP in exons 3, 4 and 10<br>• 49 SNPs at non-splice sites<br>• 2 deletions
Oca2	Chr7: 63.791 Mb (56239760-56536517 bp) on the plus strand	12.5 (1-2 months, Figure 5F); Chr7: 60.128 Mb	1418211_at; 11.87	rs13479251-59.660 Mb, rs4137599-62.761 Mb	10.186	• 3 SNPs in exon 1<br>• 5 each 3' UTR SNPs<br>• 1 each non-synonymous SNPs in exons 23 and 24<br>• 960 SNPs at non-splice sites<br>• 12 insertions<br>• 13 deletions
Myo5a	Chr9: 75.065 Mb (75071015-75223688 bp) on the plus strand	10.6 (1-2 months, Figure 5F); Chr9: 74.735 Mb	1431320_a_at; 12.5	rs13480279- 74.382 Mb, rs3725904- 76.983 Mb	8.938	• 25 SNPs at non-splice sites<br>• 17 insertions<br>• 18 deletions
Prkcz	Chr4: 154.634 Mb (155260120-155361391 bp) on the minus strand	12.3 (1-2 months, Figure 5F); Chr4:155.23 Mb	1454902_at; 1.2	rs13478070-155.04 Mb, rs3720634-155.28 Mb	8.98	• 1 non-synonymous SNP in exon 7<br>• 1 3'UTR SNP<br>• 9 SNPs at non-splice sites
Zbtb20	Chr16: 43.623 Mb (43247284-43619128 bp) on the plus strand	10.7 (3-5 months, Figure 5G); Chr16: 41.688 Mb	1439278_at; 11.7	rs4178244-41.340 Mb, rs4179378-43.943 Mb	10.719	• 84 SNPs at non-splice sites<br>• 3 non-synonymous exons in exon 8<br>• 2 insertions<br>• 2 deletions
