# Supplementary material for: Genetic modulation of the iris transillumination defect: a systems genetics analysis using the expanded family of BXD glaucoma strains
Source: Pigment Cell Melanoma Res. 2013 Apr 13;26(4):487–98. doi: 10.1111/pcmr.12106 (PMC3752936; doi:10.1111/pcmr.12106)
Supplement: Supplementary file 2 [file pcmr0026-0487-SD2.pdf]

Appendix 2: List of SNPs within *Tyrp1*

SNP ID	Mb	ConScore	Domain 1	Domain 2	Function	Details	B6	D2
wt37-4-80480728	80.480728	1	Intron	Nonsplice Site			A	G
wt37-4-80480729	80.480729	1	Intron	Nonsplice Site			G	A
wt37-4-80480743	80.480743	1	Intron	Nonsplice Site			C	A
wt37-4-80481306	80.481306	1	Exon 2	Coding	Nonsynonymous	Biotype: Protein Coding, C -> Y, tGt -> tAt, 110	G	A
wt37-4-80481565	80.481565		Intron	Nonsplice Site			G	A
wt37-4-80481748	80.481748	0.343	Intron	Nonsplice Site			T	C
wt37-4-80481902	80.481902	0.343	Intron	Nonsplice Site			C	A
wt37-4-80482143	80.482143	0.343	Intron	Nonsplice Site			G	A
wt37-4-80482194	80.482194	0.343	Intron	Nonsplice Site			T	G
wt37-4-80482339	80.482339	0.343	Intron	Nonsplice Site			A	G
MRS1284850	80.482346	0.343	Intron	Nonsplice Site			T	A
wt37-4-80482360	80.48236	0.343	Intron	Nonsplice Site			A	G
wt37-4-80482396	80.482396	0.343	Intron	Nonsplice Site			A	G
wt37-4-80485070	80.48507	1	Intron	Nonsplice Site			G	A
wt37-4-80485287	80.485287	1	Intron	Nonsplice Site			G	A
wt37-4-80485722	80.485722	1	Intron	Nonsplice Site			C	A
wt37-4-80486306	80.486306	1	Intron	Nonsplice Site			G	A
wt37-4-80486611	80.486611	1	Exon 4	Coding	Synonymous	Biotype: Protein Coding, F -> F, ttC -> ttT, 272	C	T
wt37-4-80486799	80.486799	1	Intron	Nonsplice Site			T	C
MRS1284859	80.486864	1	Intron	Nonsplice Site			T	A
wt37-4-80486922	80.486922	1	Intron	Nonsplice Site			T	G
wt37-4-80487104	80.487104	1	Intron	Nonsplice Site			A	G
wt37-4-80487370	80.48737	1	Intron	Nonsplice Site			T	G
wt37-4-80487386	80.487386	1	Intron	Nonsplice Site			C	G
wt37-4-80487428	80.487428	1	Intron	Nonsplice Site			A	T
wt37-4-80487574	80.487574	1	Intron	Nonsplice Site			T	C

wt37-4-80487575	80.487575	1	Intron	Nonsplice Site			C	T
MRS1284865	80.487578	1	Intron	Nonsplice Site			G	C
wt37-4-80487618	80.487618	0.766	Intron	Nonsplice Site			A	G
wt37-4-80487620	80.48762	0.766	Intron	Nonsplice Site			G	A
wt37-4-80487622	80.487622	0.766	Intron	Nonsplice Site			A	G
wt37-4-80487635	80.487635	0.766	Intron	Nonsplice Site			C	T
wt37-4-80487666	80.487666	0.766	Intron	Nonsplice Site			C	T
wt37-4-80487817	80.487817	0.766	Intron	Nonsplice Site			T	C
wt37-4-80488036	80.488036	0.14	Intron	Nonsplice Site			C	G
wt37-4-80488654	80.488654	0.84	Intron	Nonsplice Site			G	A
wt37-4-80489069	80.489069	0.84	Intron	Nonsplice Site			C	T
wt37-4-80490181	80.490181	0.972	Intron	Nonsplice Site			A	G
wt37-4-80490659	80.490659	1	Intron	Nonsplice Site			A	G
wt37-4-80490758	80.490758	1	Exon 5	Coding	Nonsynonymous	Biotype: Protein Coding, R -> H, cGt -> cAt, 326	G	A
wt37-4-80491480	80.49148	1	Intron	Nonsplice Site			C	T
wt37-4-80492644	80.492644	1	Exon 6	Coding	Synonymous	Biotype: Protein Coding, R -> R, agG -> agA, 417	G	A
wt37-4-80492768	80.492768	0.993	Intron	Nonsplice Site			T	G
MRS1284879	80.49302	0.993	Intron	Nonsplice Site			A	G
wt37-4-80493240	80.49324	0.993	Intron	Nonsplice Site			G	C
wt37-4-80493631	80.493631	0.187	Intron	Nonsplice Site			T	A
wt37-4-80494781	80.494781	1	Intron	Nonsplice Site			G	A
wt37-4-80495077	80.495077	1	Intron	Nonsplice Site			T	A
wt37-4-80495422	80.495422	1	Intron	Nonsplice Site			T	C
wt37-4-80496370	80.49637	1	Intron	Nonsplice Site			G	A
wt37-4-80496794	80.496794	1	Exon 3	Coding	Nonsynonymous	Biotype: Protein Coding, R -> H, cGc -> cAc, 140	G	A
wt37-4-80496948	80.496948	1	Exon	3' UTR			T	C
wt37-4-80497020	80.49702	1	Exon	3' UTR			T	G
