# Supplementary material for: Genetic modulation of the iris transillumination defect: a systems genetics analysis using the expanded family of BXD glaucoma strains
Source: Pigment Cell Melanoma Res. 2013 Apr 13;26(4):487–98. doi: 10.1111/pcmr.12106 (PMC3752936; doi:10.1111/pcmr.12106)
Supplement: Supplementary file 3 [file pcmr0026-0487-SD3.pdf]

Appendix 3: List of SNPs within *Gpnmb*

SNP ID	Mb	ConScore	Domain 1	Domain 2	Function	Details	B6	D2
wt37-6-48987092	48.987092	1	Intron	Nonsplice Site			C	T
wt37-6-48987483	48.987483	1	Intron	Nonsplice Site			G	A
wt37-6-48987502	48.987502	1	Intron	Nonsplice Site			T	C
wt37-6-48987659	48.987659	1	Intron	Nonsplice Site			T	C
wt37-6-48988396	48.988396	0.786	Intron	Nonsplice Site			C	A
wt37-6-48988911	48.988911	0.786	Intron	Nonsplice Site			T	C
wt37-6-48988953	48.988953	0.786	Intron	Nonsplice Site			G	T
wt37-6-48989072	48.989072	0.786	Intron	Nonsplice Site			C	T
wt37-6-48989074	48.989074	0.786	Intron	Nonsplice Site			A	T
wt37-6-48989302	48.989302	0.786	Intron	Nonsplice Site			A	G
wt37-6-48990254	48.990254	0.936	Intron	Nonsplice Site			C	T
wt37-6-48990837	48.990837	0.422	Intron	Nonsplice Site			A	G
wt37-6-48991322	48.991322		Intron	Nonsplice Site			A	G
wt37-6-48991336	48.991336		Intron	Nonsplice Site			A	T
wt37-6-48991901	48.991901	0.202	Intron	Nonsplice Site			T	C
wt37-6-48992050	48.99205	0.202	Intron	Nonsplice Site			G	A
wt37-6-48992571	48.992571	1	Intron	Nonsplice Site			A	G
wt37-6-48992801	48.992801	1	Exon 2	Coding	Nonsynonymous	Biotype: Protein Coding, D -> N, Gat -> Aat, 36	G	A
wt37-6-48993476	48.993476	1	Intron	Nonsplice Site			G	A
wt37-6-48993742	48.993742	1	Intron	Nonsplice Site			C	T
wt37-6-48993788	48.993788	1	Intron	Nonsplice Site			G	T
wt37-6-48993792	48.993792	1	Intron	Nonsplice Site			T	G
wt37-6-48994057	48.994057	1	Exon 3	Coding	Synonymous	Biotype: Protein Coding, T -> T, acT -> acC, 95	T	C
wt37-6-48994496	48.994496	1	Intron	Nonsplice Site			T	C
wt37-6-48994558	48.994558	1	Intron	Nonsplice Site			T	C
wt37-6-48994588	48.994588	1	Intron	Nonsplice Site			A	C
wt37-6-48995223	48.995223	1	Intron	Nonsplice Site			G	A

MRS1748503	48.995317	1	Exon 4	Coding	Synonymous	Biotype: Protein Coding, T -> T, acA -> acC, 127	A	C
wt37-6-48995384	48.995384	1	Exon 4	Coding	Stop Gained	Biotype: Protein Coding, R -> *, Cga -> Tga, 150	C	T
MRS1748505	48.995534	1	Intron	Nonsplice Site			T	G
wt37-6-48996593	48.996593	1	Intron	Nonsplice Site			A	C
MRS1748507	48.996667	1	Intron	Nonsplice Site			C	A
wt37-6-48998592	48.998592	0.467	Intron	Nonsplice Site			G	A
wt37-6-48999520	48.99952		Intron	Nonsplice Site			T	G
wt37-6-49000199	49.000199		Intron	Nonsplice Site			A	G
wt37-6-49001157	49.001157	1	Intron	Nonsplice Site			A	G
wt37-6-49002202	49.002202	1	Intron	Nonsplice Site			G	A
wt37-6-49002349	49.002349	0.971	Intron	Nonsplice Site			C	G
wt37-6-49002626	49.002626	0.971	Intron	Nonsplice Site			A	G
wt37-6-49003562	49.003562	0.901	Intron	Nonsplice Site			A	C
MRS1748517	49.004565		Intron	Nonsplice Site			G	A
wt37-6-49004930	49.00493	0.557	Intron	Nonsplice Site			G	A
wt37-6-49005229	49.005229	0.557	Intron	Nonsplice Site			A	G
wt37-6-49005666	49.005666	1	Exon 10	Coding	Synonymous	Biotype: Protein Coding, I -> I, atC -> atT, 508	C	T
wt37-6-49005885	49.005885	1	Intron	Nonsplice Site			A	G
wt37-6-49006217	49.006217	1	Intron	Nonsplice Site			G	A
wt37-6-49006238	49.006238	1	Intron	Nonsplice Site			T	C
wt37-6-49006774	49.006774	0.845	Intron	Nonsplice Site			C	T
wt37-6-49006860	49.00686	0.845	Intron	Nonsplice Site			T	C
wt37-6-49007039	49.007039	0.845	Intron	Nonsplice Site			G	T
rs38586314	49.007541		Intron	Nonsplice Site			A	T
wt37-6-49007597	49.007597	0.845	Intron	Nonsplice Site			A	C
wt37-6-49007893	49.007893	0.998	Intron	Nonsplice Site			A	G
wt37-6-49007904	49.007904	0.998	Intron	Nonsplice Site			A	G
