# Supplementary material for: Genetic modulation of the iris transillumination defect: a systems genetics analysis using the expanded family of BXD glaucoma strains
Source: Pigment Cell Melanoma Res. 2013 Apr 13;26(4):487–98. doi: 10.1111/pcmr.12106 (PMC3752936; doi:10.1111/pcmr.12106)
Supplement: Supplementary file 4 [file pcmr0026-0487-SD4.pdf]

Appendix 4: List of SNPs within *Oca2*

SNP ID	Mb	ConScore	Domain 1	Domain 2	Function	Details	B6	D2
wt37-7-63495170	63.49517	0.659	Exon 1		Unknown	Biotype: Protein Coding, Coding Region Unknown	G	A
wt37-7-63495200	63.4952	0.659	Exon 1		Unknown	Biotype: Protein Coding, Coding Region Unknown	T	G
wt37-7-63495220	63.49522	0.659	Exon 1		Unknown	Biotype: Protein Coding, Coding Region Unknown	T	C
wt37-7-63495447	63.495447	0.659	Intron	Nonsplice Site			G	A
wt37-7-63495466	63.495466	0.659	Intron	Nonsplice Site			A	C
wt37-7-63495937	63.495937	0.95	Intron	Nonsplice Site			C	T
wt37-7-63495990	63.49599	0.95	Intron	Nonsplice Site			C	G
wt37-7-63496003	63.496003	0.95	Intron	Nonsplice Site			T	C
wt37-7-63496217	63.496217	0.95	Intron	Nonsplice Site			T	C
wt37-7-63497502	63.497502	0.143	Intron	Nonsplice Site			T	C
wt37-7-63497590	63.49759	0.773	Intron	Nonsplice Site			C	T
wt37-7-63497760	63.49776	0.773	Intron	Nonsplice Site			G	A
wt37-7-63497835	63.497835	0.773	Intron	Nonsplice Site			A	G
wt37-7-63497910	63.49791	0.773	Intron	Nonsplice Site			T	C
wt37-7-63497924	63.497924	0.773	Intron	Nonsplice Site			T	G
wt37-7-63497925	63.497925	0.773	Intron	Nonsplice Site			C	A
wt37-7-63497948	63.497948	0.773	Intron	Nonsplice Site			G	A
wt37-7-63498136	63.498136	0.773	Intron	Nonsplice Site			T	C
wt37-7-63498202	63.498202	0.773	Intron	Nonsplice Site			T	C
wt37-7-63498290	63.49829	0.773	Intron	Nonsplice Site			T	C
wt37-7-63498292	63.498292	0.773	Intron	Nonsplice Site			C	A
wt37-7-63498386	63.498386	0.773	Intron	Nonsplice Site			T	A
wt37-7-63498444	63.498444	0.773	Intron	Nonsplice Site			T	C
wt37-7-63498471	63.498471	0.773	Intron	Nonsplice Site			T	C
wt37-7-63498498	63.498498	0.773	Intron	Nonsplice Site			T	C
wt37-7-63499260	63.49926		Intron	Nonsplice Site			A	G

wt37-7-63500125	63.500125		Intron	Nonsplice Site			A	G
wt37-7-63500468	63.500468	0.45	Intron	Nonsplice Site			T	C
wt37-7-63500488	63.500488	0.45	Intron	Nonsplice Site			G	T
wt37-7-63500600	63.5006	0.45	Intron	Nonsplice Site			G	C
wt37-7-63500607	63.500607	0.45	Intron	Nonsplice Site			T	A
MRS2085401	63.501238	0.45	Intron	Nonsplice Site			G	A
wt37-7-63501366	63.501366	1	Intron	Nonsplice Site			C	A
wt37-7-63501399	63.501399	1	Intron	Nonsplice Site			C	A
wt37-7-63502939	63.502939	0.998	Intron	Nonsplice Site			T	A
wt37-7-63504708	63.504708		Intron	Nonsplice Site			A	C
wt37-7-63505448	63.505448		Intron	Nonsplice Site			A	G
wt37-7-63505833	63.505833		Intron	Nonsplice Site			C	T
wt37-7-63506160	63.50616	0.434	Intron	Nonsplice Site			T	C
wt37-7-63506190	63.50619	0.434	Intron	Nonsplice Site			T	A
wt37-7-63506237	63.506237	0.434	Intron	Nonsplice Site			A	G
wt37-7-63506315	63.506315	0.434	Intron	Nonsplice Site			T	G
wt37-7-63506623	63.506623	0.434	Intron	Nonsplice Site			G	A
wt37-7-63507150	63.50715	0.612	Intron	Nonsplice Site			A	T
wt37-7-63507415	63.507415		Intron	Nonsplice Site			T	A
wt37-7-63508528	63.508528		Intron	Nonsplice Site			T	C
wt37-7-63513844	63.513844	1	Intron	Nonsplice Site			C	A
wt37-7-63514387	63.514387		Intron	Nonsplice Site			G	C
wt37-7-63514410	63.51441		Intron	Nonsplice Site			C	T
wt37-7-63514415	63.514415		Intron	Nonsplice Site			C	G
wt37-7-63514419	63.514419		Intron	Nonsplice Site			C	G
wt37-7-63514423	63.514423		Intron	Nonsplice Site			C	G
wt37-7-63514645	63.514645		Intron	Nonsplice Site			G	A

wt37-7-63514783	63.514783		Intron	Nonsplice Site			C	T
wt37-7-63514849	63.514849		Intron	Nonsplice Site			A	C
wt37-7-63514937	63.514937		Intron	Nonsplice Site			T	A
rs32201766	63.515568		Intron	Nonsplice Site			T	C
wt37-7-63515603	63.515603	0.857	Intron	Nonsplice Site			T	C
wt37-7-63515704	63.515704	0.857	Intron	Nonsplice Site			T	C
wt37-7-63515761	63.515761	0.857	Intron	Nonsplice Site			A	T
wt37-7-63515774	63.515774	0.857	Intron	Nonsplice Site			T	C
wt37-7-63515778	63.515778	0.857	Intron	Nonsplice Site			T	C
wt37-7-63515965	63.515965	0.857	Intron	Nonsplice Site			G	T
wt37-7-63515982	63.515982	0.857	Intron	Nonsplice Site			G	T
wt37-7-63516041	63.516041	0.857	Intron	Nonsplice Site			G	C
wt37-7-63516044	63.516044	0.857	Intron	Nonsplice Site			T	C
wt37-7-63516073	63.516073	0.857	Intron	Nonsplice Site			T	G
wt37-7-63516105	63.516105	0.857	Intron	Nonsplice Site			A	G
wt37-7-63516144	63.516144	0.857	Intron	Nonsplice Site			C	A
wt37-7-63516166	63.516166	0.857	Intron	Nonsplice Site			G	A
wt37-7-63516248	63.516248	0.857	Intron	Nonsplice Site			C	T
wt37-7-63516690	63.51669	0.736	Intron	Nonsplice Site			A	C
wt37-7-63516768	63.516768	0.736	Intron	Nonsplice Site			G	A
wt37-7-63517199	63.517199	0.736	Intron	Nonsplice Site			G	T
wt37-7-63517430	63.51743	0.736	Intron	Nonsplice Site			T	C
wt37-7-63517445	63.517445	0.736	Intron	Nonsplice Site			T	C
wt37-7-63517446	63.517446	0.736	Intron	Nonsplice Site			T	C
wt37-7-63517484	63.517484	0.736	Intron	Nonsplice Site			C	T
wt37-7-63517598	63.517598	0.036	Intron	Nonsplice Site			A	G
wt37-7-63517679	63.517679	0.011	Intron	Nonsplice Site			A	G

wt37-7-63517779	63.517779	0.269	Intron	Nonsplice Site			T	A
rs32977478	63.518016		Intron	Nonsplice Site			A	G
wt37-7-63518089	63.518089	0.269	Intron	Nonsplice Site			G	A
wt37-7-63518254	63.518254	0.269	Intron	Nonsplice Site			C	A
wt37-7-63518384	63.518384	0.269	Intron	Nonsplice Site			G	A
wt37-7-63518403	63.518403	0.269	Intron	Nonsplice Site			G	C
wt37-7-63518417	63.518417	0.269	Intron	Nonsplice Site			T	C
wt37-7-63518436	63.518436	0.269	Intron	Nonsplice Site			C	T
wt37-7-63518792	63.518792		Intron	Nonsplice Site			C	G
wt37-7-63518850	63.51885		Intron	Nonsplice Site			A	G
wt37-7-63518903	63.518903	0.788	Intron	Nonsplice Site			G	A
wt37-7-63518908	63.518908	0.788	Intron	Nonsplice Site			G	A
wt37-7-63518909	63.518909	0.788	Intron	Nonsplice Site			A	C
wt37-7-63518937	63.518937	0.788	Intron	Nonsplice Site			C	T
wt37-7-63519107	63.519107	0.788	Intron	Nonsplice Site			C	T
wt37-7-63519300	63.5193	0.788	Intron	Nonsplice Site			G	A
wt37-7-63519318	63.519318	0.788	Intron	Nonsplice Site			T	C
wt37-7-63519333	63.519333	0.788	Intron	Nonsplice Site			T	C
wt37-7-63519452	63.519452	0.788	Intron	Nonsplice Site			C	A
wt37-7-63519628	63.519628	0.788	Intron	Nonsplice Site			A	G
wt37-7-63519651	63.519651	0.788	Intron	Nonsplice Site			A	C
wt37-7-63519803	63.519803		Intron	Nonsplice Site			C	T
wt37-7-63519992	63.519992	0.425	Intron	Nonsplice Site			C	T
wt37-7-63520195	63.520195		Intron	Nonsplice Site			C	T
wt37-7-63520412	63.520412		Intron	Nonsplice Site			G	A
wt37-7-63520531	63.520531		Intron	Nonsplice Site			A	T
wt37-7-63520532	63.520532		Intron	Nonsplice Site			A	T

wt37-7-63520617	63.520617		Intron	Nonsplice Site			T	C
MRS2085463	63.520707		Intron	Nonsplice Site			T	C
MRS2085464	63.520763		Intron	Nonsplice Site			G	A
wt37-7-63520770	63.52077		Intron	Nonsplice Site			T	C
MRS2085466	63.520917		Intron	Nonsplice Site			A	C
MRS2085467	63.520931		Intron	Nonsplice Site			G	C
MRS2085468	63.521166		Intron	Nonsplice Site			A	G
MRS2085469	63.52128		Intron	Nonsplice Site			C	G
MRS2085470	63.521333		Intron	Nonsplice Site			A	G
MRS2085471	63.521423		Intron	Nonsplice Site			C	A
MRS2085472	63.521432		Intron	Nonsplice Site			G	A
MRS2085473	63.521491		Intron	Nonsplice Site			A	C
MRS2085474	63.52179		Intron	Nonsplice Site			A	C
MRS2085475	63.52192		Intron	Nonsplice Site			T	G
MRS2085476	63.521934		Intron	Nonsplice Site			A	G
MRS2085477	63.52199		Intron	Nonsplice Site			T	C
MRS2085478	63.522005		Intron	Nonsplice Site			A	T
MRS2085479	63.522134		Intron	Nonsplice Site			G	C
MRS2085480	63.522529		Intron	Nonsplice Site			G	T
MRS2085481	63.522597		Intron	Nonsplice Site			G	T
MRS2085482	63.522639		Intron	Nonsplice Site			T	C
MRS2085483	63.522698		Intron	Nonsplice Site			T	C
wt37-7-63522780	63.52278		Intron	Nonsplice Site			G	T
MRS2085486	63.523028		Intron	Nonsplice Site			C	T
MRS2085487	63.523252		Intron	Nonsplice Site			T	C
MRS2085488	63.523319		Intron	Nonsplice Site			C	T
MRS2085489	63.523332		Intron	Nonsplice Site			T	C

MRS2085490	63.523356		Intron	Nonsplice Site			G	T
wt37-7-63523627	63.523627		Intron	Nonsplice Site			C	T
MRS2085491	63.523727		Intron	Nonsplice Site			C	T
MRS2085492	63.52383		Intron	Nonsplice Site			G	A
MRS2085493	63.523835		Intron	Nonsplice Site			A	T
MRS2085494	63.523968		Intron	Nonsplice Site			G	T
MRS2085495	63.524043		Intron	Nonsplice Site			A	G
MRS2085496	63.524112		Intron	Nonsplice Site			C	T
MRS2085497	63.524238		Intron	Nonsplice Site			C	T
MRS2085498	63.52427		Intron	Nonsplice Site			G	C
MRS2085499	63.524432		Intron	Nonsplice Site			C	G
MRS2085500	63.524451		Intron	Nonsplice Site			G	A
MRS2085501	63.524776		Intron	Nonsplice Site			G	C
MRS2085502	63.524806		Intron	Nonsplice Site			C	G
MRS2085503	63.525003		Intron	Nonsplice Site			T	A
MRS2085504	63.525072		Intron	Nonsplice Site			T	C
MRS2085505	63.525368		Intron	Nonsplice Site			C	T
MRS2085506	63.525502		Intron	Nonsplice Site			C	A
MRS2085507	63.525551		Intron	Nonsplice Site			A	G
MRS2085508	63.525694		Intron	Nonsplice Site			C	T
MRS2085509	63.527178		Intron	Nonsplice Site			A	G
wt37-7-63531344	63.531344	0.409	Intron	Nonsplice Site			G	A
wt37-7-63531345	63.531345	0.409	Intron	Nonsplice Site			A	T
wt37-7-63531777	63.531777	0.494	Intron	Nonsplice Site			C	T
MRS2085511	63.532144	0.494	Intron	Nonsplice Site			T	G
wt37-7-63534559	63.534559		Intron	Nonsplice Site			G	C
wt37-7-63534896	63.534896	0.678	Intron	Nonsplice Site			A	G

MRS2085514	63.534897	0.678	Intron	Nonsplice Site			C	T
wt37-7-63534898	63.534898	0.678	Intron	Nonsplice Site			T	C
wt37-7-63534930	63.53493	0.678	Intron	Nonsplice Site			A	C
wt37-7-63535918	63.535918	0.919	Intron	Nonsplice Site			C	T
wt37-7-63536229	63.536229	0.919	Exon	3' UTR			T	A
wt37-7-63536469	63.536469	0.919	Intron	Nonsplice Site			A	T
wt37-7-63536500	63.5365	0.919	Intron	Nonsplice Site			T	A
wt37-7-63536541	63.536541	0.919	Intron	Nonsplice Site			C	T
wt37-7-63536564	63.536564	0.919	Intron	Nonsplice Site			C	T
wt37-7-63536569	63.536569	0.919	Intron	Nonsplice Site			G	A
MRS2085523	63.537655	0.851	Intron	Nonsplice Site			G	A
wt37-7-63537656	63.537656	0.851	Intron	Nonsplice Site			G	A
wt37-7-63538039	63.538039	0.965	Intron	Nonsplice Site			G	A
wt37-7-63538052	63.538052	0.965	Intron	Nonsplice Site			G	A
wt37-7-63538288	63.538288	0.965	Intron	Nonsplice Site			T	C
wt37-7-63538291	63.538291	0.965	Intron	Nonsplice Site			A	G
wt37-7-63539044	63.539044	0.929	Intron	Nonsplice Site			T	C
wt37-7-63539230	63.53923	0.929	Intron	Nonsplice Site			T	G
wt37-7-63540973	63.540973	0.515	Intron	Nonsplice Site			A	G
wt37-7-63541455	63.541455	0.515	Intron	Nonsplice Site			C	T
wt37-7-63541781	63.541781		Intron	Nonsplice Site			T	A
wt37-7-63542650	63.54265	0.503	Intron	Nonsplice Site			C	T
wt37-7-63542739	63.542739	0.503	Intron	Nonsplice Site			T	C
wt37-7-63542824	63.542824	0.503	Intron	Nonsplice Site			C	A
wt37-7-63542919	63.542919	0.503	Intron	Nonsplice Site			A	G
wt37-7-63542991	63.542991	0.503	Intron	Nonsplice Site			C	T
wt37-7-63543105	63.543105	0.503	Intron	Nonsplice Site			A	T

wt37-7-63543240	63.54324		Intron	Nonsplice Site			C	T
wt37-7-63543321	63.543321		Intron	Nonsplice Site			T	C
wt37-7-63543556	63.543556	0.781	Intron	Nonsplice Site			A	G
wt37-7-63543658	63.543658	0.781	Intron	Nonsplice Site			A	G
wt37-7-63543695	63.543695	0.781	Intron	Nonsplice Site			A	T
wt37-7-63543914	63.543914	0.781	Intron	Nonsplice Site			A	G
wt37-7-63543992	63.543992	0.781	Intron	Nonsplice Site			A	G
wt37-7-63543996	63.543996	0.781	Intron	Nonsplice Site			A	G
wt37-7-63544000	63.544	0.781	Intron	Nonsplice Site			A	G
wt37-7-63544004	63.544004	0.781	Intron	Nonsplice Site			A	G
wt37-7-63544008	63.544008	0.781	Intron	Nonsplice Site			A	G
wt37-7-63544012	63.544012	0.781	Intron	Nonsplice Site			A	G
wt37-7-63544016	63.544016	0.781	Intron	Nonsplice Site			A	G
wt37-7-63544080	63.54408	0.781	Intron	Nonsplice Site			T	C
wt37-7-63544129	63.544129	0.781	Intron	Nonsplice Site			A	C
wt37-7-63544472	63.544472	0.781	Intron	Nonsplice Site			G	T
wt37-7-63544703	63.544703	0.998	Intron	Nonsplice Site			C	G
wt37-7-63545034	63.545034	0.998	Intron	Nonsplice Site			T	C
wt37-7-63545199	63.545199	0.998	Intron	Nonsplice Site			T	C
wt37-7-63545606	63.545606	0.382	Intron	Nonsplice Site			C	T
wt37-7-63545798	63.545798	0.382	Intron	Nonsplice Site			C	T
wt37-7-63546391	63.546391	0.382	Intron	Nonsplice Site			G	C
wt37-7-63546549	63.546549	0.867	Intron	Nonsplice Site			T	C
wt37-7-63546899	63.546899	0.867	Intron	Nonsplice Site			G	A
wt37-7-63546912	63.546912	0.867	Intron	Nonsplice Site			G	T
MRS2085559	63.547367	0.867	Intron	Nonsplice Site			G	A
wt37-7-63547648	63.547648	0.895	Intron	Nonsplice Site			C	T

wt37-7-63547864	63.547864	0.895	Intron	Nonsplice Site			A	G
wt37-7-63547897	63.547897	0.895	Intron	Nonsplice Site			T	A
wt37-7-63547908	63.547908		Intron	Nonsplice Site			T	G
wt37-7-63547910	63.54791		Intron	Nonsplice Site			T	G
wt37-7-63548388	63.548388	0.579	Intron	Nonsplice Site			G	T
wt37-7-63548400	63.5484	0.579	Intron	Nonsplice Site			C	G
wt37-7-63548409	63.548409	0.579	Intron	Nonsplice Site			T	C
wt37-7-63548431	63.548431	0.579	Intron	Nonsplice Site			C	T
wt37-7-63548495	63.548495	0.579	Intron	Nonsplice Site			A	G
wt37-7-63548540	63.54854		Intron	Nonsplice Site			A	G
wt37-7-63548548	63.548548	1	Intron	Nonsplice Site			T	C
wt37-7-63548578	63.548578	1	Intron	Nonsplice Site			C	T
wt37-7-63548582	63.548582	1	Intron	Nonsplice Site			T	C
wt37-7-63548593	63.548593	1	Intron	Nonsplice Site			G	A
wt37-7-63548594	63.548594	1	Intron	Nonsplice Site			T	C
wt37-7-63548629	63.548629	1	Intron	Nonsplice Site			G	A
wt37-7-63548632	63.548632	1	Intron	Nonsplice Site			C	T
wt37-7-63548641	63.548641	1	Intron	Nonsplice Site			G	A
wt37-7-63548644	63.548644	1	Intron	Nonsplice Site			C	G
wt37-7-63548681	63.548681	1	Intron	Nonsplice Site			C	T
wt37-7-63548695	63.548695	1	Intron	Nonsplice Site			G	A
wt37-7-63548696	63.548696	1	Intron	Nonsplice Site			A	G
wt37-7-63548725	63.548725	1	Intron	Nonsplice Site			T	A
wt37-7-63548731	63.548731	1	Intron	Nonsplice Site			T	C
wt37-7-63548760	63.54876	1	Intron	Nonsplice Site			T	C
wt37-7-63548761	63.548761	1	Intron	Nonsplice Site			G	T
wt37-7-63548781	63.548781	1	Intron	Nonsplice Site			T	C

wt37-7-63548782	63.548782	1	Intron	Nonsplice Site			G	A
wt37-7-63548860	63.54886	1	Intron	Nonsplice Site			G	T
wt37-7-63548924	63.548924	1	Intron	Nonsplice Site			G	A
wt37-7-63549155	63.549155	1	Exon	3' UTR			A	T
wt37-7-63549529	63.549529	1	Intron	Nonsplice Site			C	T
wt37-7-63549578	63.549578	0.522	Intron	Nonsplice Site			A	G
wt37-7-63549589	63.549589	0.522	Intron	Nonsplice Site			T	G
wt37-7-63549684	63.549684	0.522	Intron	Nonsplice Site			C	A
wt37-7-63550944	63.550944	1	Intron	Nonsplice Site			C	T
wt37-7-63551565	63.551565	1	Intron	Nonsplice Site			T	G
wt37-7-63552076	63.552076	1	Intron	Nonsplice Site			C	T
wt37-7-63552720	63.55272	0.47	Intron	Nonsplice Site			C	G
MRS2085587	63.553755	0.393	Intron	Nonsplice Site			A	T
wt37-7-63554184	63.554184	0.716	Intron	Nonsplice Site			C	T
wt37-7-63554697	63.554697	0.716	Intron	Nonsplice Site			G	A
wt37-7-63554908	63.554908	0.716	Intron	Nonsplice Site			A	G
MRS2085591	63.555474	0.311	Intron	Nonsplice Site			C	A
wt37-7-63555879	63.555879	0.311	Intron	Nonsplice Site			G	T
wt37-7-63555891	63.555891	0.311	Intron	Nonsplice Site			T	C
wt37-7-63555918	63.555918	0.311	Intron	Nonsplice Site			A	G
wt37-7-63556460	63.55646	0.803	Intron	Nonsplice Site			T	C
wt37-7-63556570	63.55657	0.803	Intron	Nonsplice Site			G	A
wt37-7-63557205	63.557205	0.524	Intron	Nonsplice Site			C	T
wt37-7-63557664	63.557664	0.524	Intron	Nonsplice Site			C	A
wt37-7-63557828	63.557828	0.524	Intron	Nonsplice Site			A	G
wt37-7-63558641	63.558641	0.779	Intron	Nonsplice Site			A	G
wt37-7-63558891	63.558891	0.779	Intron	Nonsplice Site			G	A

wt37-7-63558979	63.558979	0.779	Intron	Nonsplice Site			C	T
wt37-7-63559272	63.559272	0.737	Intron	Nonsplice Site			A	G
wt37-7-63559582	63.559582	0.737	Intron	Nonsplice Site			T	C
wt37-7-63560110	63.56011	0.737	Intron	Nonsplice Site			G	A
wt37-7-63560887	63.560887		Intron	Nonsplice Site			G	A
wt37-7-63561396	63.561396	0.717	Intron	Nonsplice Site			G	C
wt37-7-63561741	63.561741	0.717	Intron	Nonsplice Site			T	A
wt37-7-63561865	63.561865	0.717	Intron	Nonsplice Site			G	A
wt37-7-63562227	63.562227	0.717	Intron	Nonsplice Site			C	T
wt37-7-63562339	63.562339	0.72	Intron	Nonsplice Site			G	C
wt37-7-63563258	63.563258	0.72	Intron	Nonsplice Site			G	A
wt37-7-63563320	63.56332	0.72	Intron	Nonsplice Site			T	C
wt37-7-63563337	63.563337	0.72	Intron	Nonsplice Site			T	C
wt37-7-63564113	63.564113	0.726	Intron	Nonsplice Site			G	A
wt37-7-63564264	63.564264	0.726	Intron	Nonsplice Site			C	A
wt37-7-63564276	63.564276	0.726	Intron	Nonsplice Site			C	A
wt37-7-63564278	63.564278	0.726	Intron	Nonsplice Site			C	A
wt37-7-63565045	63.565045		Intron	Nonsplice Site			C	T
wt37-7-63566270	63.56627	0.861	Intron	Nonsplice Site			T	A
wt37-7-63566286	63.566286	0.861	Intron	Nonsplice Site			C	A
wt37-7-63567015	63.567015	0.861	Intron	Nonsplice Site			A	G
wt37-7-63567661	63.567661	0.441	Intron	Nonsplice Site			G	C
wt37-7-63567864	63.567864	0.441	Intron	Nonsplice Site			G	A
wt37-7-63567913	63.567913	0.441	Intron	Nonsplice Site			T	C
wt37-7-63568079	63.568079	0.441	Intron	Nonsplice Site			G	C
wt37-7-63568084	63.568084	0.441	Intron	Nonsplice Site			C	T
wt37-7-63568427	63.568427	0.441	Intron	Nonsplice Site			G	C

wt37-7-63568647	63.568647		Intron	Nonsplice Site			C	G
wt37-7-63569085	63.569085		Intron	Nonsplice Site			G	T
wt37-7-63569317	63.569317	1	Intron	Nonsplice Site			T	C
wt37-7-63569657	63.569657	1	Intron	Nonsplice Site			C	T
wt37-7-63571035	63.571035		Intron	Nonsplice Site			A	G
wt37-7-63571036	63.571036		Intron	Nonsplice Site			G	A
wt37-7-63571084	63.571084		Intron	Nonsplice Site			A	G
wt37-7-63571265	63.571265	1	Intron	Nonsplice Site			G	A
wt37-7-63572321	63.572321		Intron	Nonsplice Site			A	T
MRS2085637	63.573013		Intron	Nonsplice Site			A	T
MRS2085638	63.573647		Intron	Nonsplice Site			A	G
MRS2085639	63.573906		Intron	Nonsplice Site			T	C
wt37-7-63574048	63.574048		Intron	Nonsplice Site			A	G
wt37-7-63574823	63.574823	0.854	Intron	Nonsplice Site			G	A
MRS2085642	63.575163	0.854	Intron	Nonsplice Site			A	C
wt37-7-63575190	63.57519	0.854	Intron	Nonsplice Site			A	C
wt37-7-63575198	63.575198	0.854	Intron	Nonsplice Site			T	A
wt37-7-63575237	63.575237	0.854	Intron	Nonsplice Site			G	A
wt37-7-63575599	63.575599	0.074	Intron	Nonsplice Site			A	G
wt37-7-63575614	63.575614	0.074	Intron	Nonsplice Site			G	A
wt37-7-63575699	63.575699	0.074	Intron	Nonsplice Site			C	G
wt37-7-63575970	63.57597	0.074	Intron	Nonsplice Site			G	A
MRS2085650	63.576083	1	Intron	Nonsplice Site			T	C
wt37-7-63576146	63.576146	1	Intron	Nonsplice Site			T	A
wt37-7-63576219	63.576219	1	Intron	Nonsplice Site			G	A
wt37-7-63576225	63.576225	1	Intron	Nonsplice Site			A	G
wt37-7-63576261	63.576261	1	Intron	Nonsplice Site			G	A

wt37-7-63576456	63.576456	1	Intron	Nonsplice Site			G	T
wt37-7-63576530	63.57653	1	Intron	Nonsplice Site			T	A
wt37-7-63576591	63.576591	1	Intron	Nonsplice Site			G	A
wt37-7-63576630	63.57663	1	Intron	Nonsplice Site			C	T
wt37-7-63576721	63.576721	1	Intron	Nonsplice Site			A	T
wt37-7-63576734	63.576734	1	Intron	Nonsplice Site			T	G
wt37-7-63576768	63.576768	1	Intron	Nonsplice Site			C	T
wt37-7-63576808	63.576808	1	Intron	Nonsplice Site			A	G
wt37-7-63576816	63.576816	1	Intron	Nonsplice Site			G	A
wt37-7-63576833	63.576833	1	Intron	Nonsplice Site			G	A
wt37-7-63576966	63.576966	1	Intron	Nonsplice Site			G	T
wt37-7-63576979	63.576979	1	Intron	Nonsplice Site			G	A
wt37-7-63577025	63.577025	1	Intron	Nonsplice Site			A	G
wt37-7-63577026	63.577026	1	Intron	Nonsplice Site			T	C
wt37-7-63577205	63.577205	0.881	Intron	Nonsplice Site			C	T
wt37-7-63577258	63.577258	0.881	Intron	Nonsplice Site			G	A
wt37-7-63577282	63.577282	0.881	Intron	Nonsplice Site			A	G
wt37-7-63577294	63.577294	0.881	Intron	Nonsplice Site			G	A
wt37-7-63577309	63.577309	0.881	Intron	Nonsplice Site			G	A
wt37-7-63577377	63.577377	0.881	Intron	Nonsplice Site			A	T
wt37-7-63577388	63.577388	0.881	Intron	Nonsplice Site			A	G
wt37-7-63577416	63.577416	0.881	Intron	Nonsplice Site			A	G
wt37-7-63577420	63.57742	0.881	Intron	Nonsplice Site			G	A
wt37-7-63577496	63.577496	0.881	Intron	Nonsplice Site			C	T
wt37-7-63577639	63.577639	0.881	Intron	Nonsplice Site			A	T
wt37-7-63578292	63.578292	0.396	Intron	Nonsplice Site			T	C
wt37-7-63578736	63.578736	1	Intron	Nonsplice Site			C	T

wt37-7-63579479	63.579479	1	Intron	Nonsplice Site			A	G
wt37-7-63579524	63.579524	1	Intron	Nonsplice Site			T	A
wt37-7-63580033	63.580033	1	Exon	3' UTR			A	G
wt37-7-63580235	63.580235	1	Intron	Nonsplice Site			A	C
wt37-7-63580847	63.580847	0.817	Intron	Nonsplice Site			G	A
wt37-7-63581090	63.58109	0.817	Intron	Nonsplice Site			A	T
wt37-7-63581094	63.581094	0.817	Intron	Nonsplice Site			C	T
wt37-7-63581240	63.58124	0.817	Intron	Nonsplice Site			C	T
wt37-7-63581253	63.581253	0.817	Intron	Nonsplice Site			A	G
wt37-7-63581367	63.581367	0.817	Intron	Nonsplice Site			T	G
wt37-7-63581450	63.58145	0.817	Intron	Nonsplice Site			G	T
wt37-7-63581549	63.581549	0.817	Intron	Nonsplice Site			C	T
wt37-7-63581553	63.581553	0.817	Intron	Nonsplice Site			T	C
wt37-7-63581603	63.581603	0.817	Intron	Nonsplice Site			A	G
wt37-7-63581660	63.58166	0.142	Intron	Nonsplice Site			C	T
wt37-7-63581690	63.58169	0.142	Intron	Nonsplice Site			T	A
wt37-7-63581842	63.581842	0.142	Intron	Nonsplice Site			C	A
wt37-7-63581867	63.581867	0.142	Intron	Nonsplice Site			G	C
wt37-7-63581878	63.581878	0.142	Intron	Nonsplice Site			A	G
wt37-7-63581894	63.581894	0.142	Intron	Nonsplice Site			C	T
wt37-7-63581908	63.581908	0.142	Intron	Nonsplice Site			C	T
wt37-7-63581910	63.58191	0.142	Intron	Nonsplice Site			A	G
wt37-7-63582087	63.582087	0.142	Intron	Nonsplice Site			T	C
wt37-7-63582094	63.582094	0.142	Intron	Nonsplice Site			G	A
wt37-7-63582157	63.582157		Intron	Nonsplice Site			T	A
wt37-7-63582184	63.582184		Intron	Nonsplice Site			A	G
wt37-7-63582197	63.582197		Intron	Nonsplice Site			A	G

wt37-7-63582214	63.582214		Intron	Nonsplice Site			A	G
wt37-7-63582240	63.58224		Intron	Nonsplice Site			T	A
wt37-7-63582244	63.582244		Intron	Nonsplice Site			G	A
wt37-7-63582254	63.582254		Intron	Nonsplice Site			C	T
wt37-7-63582269	63.582269		Intron	Nonsplice Site			C	T
wt37-7-63582393	63.582393	1	Intron	Nonsplice Site			G	T
wt37-7-63582461	63.582461	1	Intron	Nonsplice Site			A	T
wt37-7-63582544	63.582544	1	Intron	Nonsplice Site			C	T
wt37-7-63582547	63.582547	1	Intron	Nonsplice Site			T	C
wt37-7-63582593	63.582593	1	Intron	Nonsplice Site			A	C
wt37-7-63582607	63.582607	1	Intron	Nonsplice Site			T	C
wt37-7-63582635	63.582635	1	Exon	3' UTR			C	T
wt37-7-63582776	63.582776	1	Intron	Nonsplice Site			T	C
wt37-7-63582863	63.582863	1	Intron	Nonsplice Site			T	C
wt37-7-63583075	63.583075	1	Intron	Nonsplice Site			G	T
wt37-7-63583956	63.583956	1	Intron	Nonsplice Site			A	G
MRS2085723	63.585034	1	Intron	Nonsplice Site			G	A
wt37-7-63585165	63.585165	1	Intron	Nonsplice Site			A	C
wt37-7-63588026	63.588026	0.268	Intron	Nonsplice Site			T	G
wt37-7-63588431	63.588431		Intron	Nonsplice Site			A	G
wt37-7-63588466	63.588466		Intron	Nonsplice Site			C	T
wt37-7-63588610	63.58861		Intron	Nonsplice Site			G	A
wt37-7-63588680	63.58868		Intron	Nonsplice Site			A	G
wt37-7-63588729	63.588729		Intron	Nonsplice Site			T	A
wt37-7-63589740	63.58974	0.945	Intron	Nonsplice Site			A	G
wt37-7-63590162	63.590162		Intron	Nonsplice Site			T	C
wt37-7-63590299	63.590299		Intron	Nonsplice Site			G	A

wt37-7-63591050	63.59105	0.594	Intron	Nonsplice Site			T	C
wt37-7-63591491	63.591491	0.612	Intron	Nonsplice Site			G	T
wt37-7-63591685	63.591685		Intron	Nonsplice Site			C	T
wt37-7-63596929	63.596929	0.669	Intron	Nonsplice Site			C	T
wt37-7-63597332	63.597332	0.125	Intron	Nonsplice Site			A	G
MRS2085740	63.597333	0.125	Intron	Nonsplice Site			T	A
wt37-7-63597349	63.597349	0.125	Intron	Nonsplice Site			C	A
rs31250970	63.597421		Intron	Nonsplice Site			A	C
wt37-7-63597426	63.597426		Intron	Nonsplice Site			A	C
wt37-7-63597455	63.597455		Intron	Nonsplice Site			G	C
MRS2085743	63.597973		Intron	Nonsplice Site			A	G
MRS2085744	63.598532		Intron	Nonsplice Site			G	C
MRS2085745	63.599819		Intron	Nonsplice Site			A	G
MRS2085746	63.599835		Intron	Nonsplice Site			G	T
MRS2085747	63.599878		Intron	Nonsplice Site			C	A
MRS2085748	63.60017		Intron	Nonsplice Site			T	C
MRS2085749	63.600617		Intron	Nonsplice Site			G	A
MRS2085750	63.600623		Intron	Nonsplice Site			C	G
MRS2085751	63.600749		Intron	Nonsplice Site			C	T
MRS2085752	63.600764		Intron	Nonsplice Site			A	G
wt37-7-63600807	63.600807		Intron	Nonsplice Site			T	C
MRS2085754	63.600983		Intron	Nonsplice Site			C	T
MRS2085755	63.601308		Intron	Nonsplice Site			T	C
MRS2085756	63.601851		Intron	Nonsplice Site			C	T
MRS2085757	63.602695		Intron	Nonsplice Site			C	T
MRS2085758	63.602783		Intron	Nonsplice Site			A	C
wt37-7-63603427	63.603427		Intron	Nonsplice Site			C	T

wt37-7-63603823	63.603823	0.967	Intron	Nonsplice Site			G	A
wt37-7-63603870	63.60387	0.967	Intron	Nonsplice Site			T	C
wt37-7-63604121	63.604121	0.967	Intron	Nonsplice Site			G	C
wt37-7-63604412	63.604412	0.967	Intron	Nonsplice Site			C	G
wt37-7-63604707	63.604707	0.855	Intron	Nonsplice Site			G	A
wt37-7-63605413	63.605413	0.855	Intron	Nonsplice Site			C	T
wt37-7-63606239	63.606239	0.823	Intron	Nonsplice Site			T	C
wt37-7-63606377	63.606377	0.823	Intron	Nonsplice Site			G	C
wt37-7-63606725	63.606725	0.297	Intron	Nonsplice Site			T	A
wt37-7-63606884	63.606884	0.297	Intron	Nonsplice Site			T	C
wt37-7-63607291	63.607291		Intron	Nonsplice Site			T	G
wt37-7-63608074	63.608074		Intron	Nonsplice Site			A	T
wt37-7-63608353	63.608353	0.714	Intron	Nonsplice Site			A	G
wt37-7-63608520	63.60852	0.714	Intron	Nonsplice Site			A	G
wt37-7-63608559	63.608559	0.714	Intron	Nonsplice Site			A	G
wt37-7-63608642	63.608642	0.714	Intron	Nonsplice Site			A	C
wt37-7-63609254	63.609254	0.714	Intron	Nonsplice Site			G	T
wt37-7-63609767	63.609767	0.902	Intron	Nonsplice Site			T	C
wt37-7-63611138	63.611138	0.39	Intron	Nonsplice Site			T	G
wt37-7-63611395	63.611395	0.39	Intron	Nonsplice Site			T	C
wt37-7-63611974	63.611974	0.515	Intron	Nonsplice Site			T	C
wt37-7-63612267	63.612267	0.515	Intron	Nonsplice Site			C	T
wt37-7-63612461	63.612461	1	Intron	Nonsplice Site			T	C
wt37-7-63613037	63.613037	1	Intron	Nonsplice Site			C	T
wt37-7-63613379	63.613379	1	Intron	Nonsplice Site			C	T
wt37-7-63613535	63.613535	0.453	Intron	Nonsplice Site			T	C
wt37-7-63613565	63.613565	0.453	Intron	Nonsplice Site			G	C

wt37-7-63613577	63.613577	0.453	Intron	Nonsplice Site			G	C
wt37-7-63613579	63.613579	0.453	Intron	Nonsplice Site			C	G
wt37-7-63614566	63.614566		Intron	Nonsplice Site			G	A
wt37-7-63614606	63.614606		Intron	Nonsplice Site			T	G
wt37-7-63617267	63.617267		Intron	Nonsplice Site			T	C
wt37-7-63617544	63.617544	0.672	Intron	Nonsplice Site			G	A
wt37-7-63619041	63.619041		Intron	Nonsplice Site			A	G
wt37-7-63620848	63.620848		Intron	Nonsplice Site			C	T
wt37-7-63620873	63.620873		Intron	Nonsplice Site			C	A
wt37-7-63620899	63.620899		Intron	Nonsplice Site			T	C
wt37-7-63620906	63.620906		Intron	Nonsplice Site			C	T
wt37-7-63620967	63.620967		Intron	Nonsplice Site			C	T
rs32438536	63.621177		Intron	Nonsplice Site			G	T
MRS2085797	63.62119		Intron	Nonsplice Site			C	T
wt37-7-63621208	63.621208		Intron	Nonsplice Site			C	T
wt37-7-63621346	63.621346	0.699	Intron	Nonsplice Site			C	T
wt37-7-63621407	63.621407	0.699	Intron	Nonsplice Site			A	G
wt37-7-63621463	63.621463	0.699	Intron	Nonsplice Site			A	G
wt37-7-63621484	63.621484	0.699	Intron	Nonsplice Site			A	T
wt37-7-63621545	63.621545	0.699	Intron	Nonsplice Site			T	C
wt37-7-63621562	63.621562	0.699	Intron	Nonsplice Site			T	C
wt37-7-63621584	63.621584	0.699	Intron	Nonsplice Site			T	C
wt37-7-63621646	63.621646	0.699	Intron	Nonsplice Site			A	G
MRS2085807	63.621663	0.699	Intron	Nonsplice Site			C	A
wt37-7-63621687	63.621687	0.699	Intron	Nonsplice Site			T	C
wt37-7-63621734	63.621734		Intron	Nonsplice Site			C	T
wt37-7-63621738	63.621738		Intron	Nonsplice Site			C	T

wt37-7-63621791	63.621791		Intron	Nonsplice Site			T	C
wt37-7-63621806	63.621806	0.783	Intron	Nonsplice Site			T	C
wt37-7-63621832	63.621832	0.783	Intron	Nonsplice Site			A	G
wt37-7-63621844	63.621844	0.783	Intron	Nonsplice Site			C	A
wt37-7-63621871	63.621871	0.783	Intron	Nonsplice Site			C	T
wt37-7-63622072	63.622072	0.783	Intron	Nonsplice Site			T	G
wt37-7-63622107	63.622107	0.783	Intron	Nonsplice Site			G	A
wt37-7-63622140	63.62214	0.783	Intron	Nonsplice Site			C	A
wt37-7-63622246	63.622246	0.783	Intron	Nonsplice Site			T	C
wt37-7-63622257	63.622257	0.783	Intron	Nonsplice Site			C	T
wt37-7-63622440	63.62244	0.783	Intron	Nonsplice Site			G	A
wt37-7-63622681	63.622681	0.783	Intron	Nonsplice Site			A	C
wt37-7-63622763	63.622763	0.783	Intron	Nonsplice Site			A	G
wt37-7-63623098	63.623098	0.104	Intron	Nonsplice Site			C	T
wt37-7-63623168	63.623168		Intron	Nonsplice Site			T	C
wt37-7-63623398	63.623398	0.636	Intron	Nonsplice Site			T	C
wt37-7-63623446	63.623446	0.636	Intron	Nonsplice Site			T	G
wt37-7-63623554	63.623554	0.636	Intron	Nonsplice Site			C	T
wt37-7-63623596	63.623596	0.636	Intron	Nonsplice Site			C	T
wt37-7-63623641	63.623641	0.636	Intron	Nonsplice Site			A	G
wt37-7-63624063	63.624063	0.636	Intron	Nonsplice Site			A	G
wt37-7-63624084	63.624084		Intron	Nonsplice Site			A	T
wt37-7-63624112	63.624112		Intron	Nonsplice Site			A	T
wt37-7-63624129	63.624129		Intron	Nonsplice Site			C	T
wt37-7-63624137	63.624137		Intron	Nonsplice Site			C	T
wt37-7-63624569	63.624569	0.908	Intron	Nonsplice Site			C	T
wt37-7-63624577	63.624577	0.908	Intron	Nonsplice Site			C	A

wt37-7-63624579	63.624579	0.908	Intron	Nonsplice Site			T	C
wt37-7-63624713	63.624713	0.908	Intron	Nonsplice Site			A	G
wt37-7-63625020	63.62502	0.908	Intron	Nonsplice Site			T	C
wt37-7-63625335	63.625335	0.635	Intron	Nonsplice Site			A	C
wt37-7-63625359	63.625359	0.635	Intron	Nonsplice Site			A	C
wt37-7-63625989	63.625989		Intron	Nonsplice Site			C	A
wt37-7-63626289	63.626289		Intron	Nonsplice Site			G	A
wt37-7-63626445	63.626445		Intron	Nonsplice Site			A	T
MRS2085841	63.627446		Intron	Nonsplice Site			T	A
MRS2085842	63.627912		Intron	Nonsplice Site			A	G
wt37-7-63628779	63.628779		Intron	Nonsplice Site			A	G
MRS2085844	63.629413		Intron	Nonsplice Site			G	A
wt37-7-63630080	63.63008	0.453	Intron	Nonsplice Site			C	A
wt37-7-63630360	63.63036	0.453	Intron	Nonsplice Site			C	T
wt37-7-63633652	63.633652		Intron	Nonsplice Site			C	G
wt37-7-63634026	63.634026		Intron	Nonsplice Site			T	C
wt37-7-63634769	63.634769		Intron	Nonsplice Site			C	T
wt37-7-63635098	63.635098		Intron	Nonsplice Site			A	G
wt37-7-63635276	63.635276		Intron	Nonsplice Site			C	T
wt37-7-63636582	63.636582		Intron	Nonsplice Site			A	G
wt37-7-63637175	63.637175		Intron	Nonsplice Site			G	A
wt37-7-63637985	63.637985	0.989	Intron	Nonsplice Site			A	T
wt37-7-63639501	63.639501	0.599	Intron	Nonsplice Site			G	A
wt37-7-63639632	63.639632		Intron	Nonsplice Site			A	C
wt37-7-63640604	63.640604	0.875	Intron	Nonsplice Site			A	G
wt37-7-63640660	63.64066	0.875	Intron	Nonsplice Site			G	T
MRS2085859	63.641132	0.875	Intron	Nonsplice Site			A	C

wt37-7-63642074	63.642074	0.524	Intron	Nonsplice Site			A	C
wt37-7-63642395	63.642395		Intron	Nonsplice Site			G	A
MRS2085862	63.642826		Intron	Nonsplice Site			G	T
wt37-7-63643038	63.643038		Intron	Nonsplice Site			A	G
wt37-7-63643994	63.643994	0.467	Intron	Nonsplice Site			A	C
wt37-7-63644733	63.644733		Intron	Nonsplice Site			T	A
wt37-7-63645110	63.64511	0.99	Intron	Nonsplice Site			C	T
wt37-7-63645240	63.64524	0.99	Intron	Nonsplice Site			T	C
wt37-7-63645865	63.645865	0.424	Intron	Nonsplice Site			C	T
wt37-7-63646326	63.646326	0.424	Intron	Nonsplice Site			T	C
wt37-7-63646533	63.646533	0.424	Intron	Nonsplice Site			C	T
wt37-7-63646571	63.646571	0.424	Intron	Nonsplice Site			A	T
wt37-7-63647777	63.647777	0.334	Intron	Nonsplice Site			A	G
wt37-7-63647984	63.647984	0.334	Intron	Nonsplice Site			A	G
rs31137281	63.648167		Intron	Nonsplice Site			A	G
wt37-7-63648190	63.64819	0.334	Intron	Nonsplice Site			A	G
wt37-7-63648553	63.648553	0.334	Intron	Nonsplice Site			G	A
wt37-7-63648695	63.648695	0.334	Intron	Nonsplice Site			A	C
rs32980581	63.648753		Intron	Nonsplice Site			A	G
MRS2085876	63.64899		Intron	Nonsplice Site			C	G
wt37-7-63649120	63.64912		Intron	Nonsplice Site			C	T
MRS2085878	63.64949		Intron	Nonsplice Site			G	A
MRS2085879	63.649509		Intron	Nonsplice Site			G	C
MRS2085880	63.649726		Intron	Nonsplice Site			G	A
MRS2085881	63.649758		Intron	Nonsplice Site			A	T
wt37-7-63650520	63.65052		Intron	Nonsplice Site			T	A
MRS2085882	63.651562		Intron	Nonsplice Site			G	A

wt37-7-63651638	63.651638		Intron	Nonsplice Site			T	C
MRS2085884	63.651708		Intron	Nonsplice Site			A	C
MRS2085885	63.651709		Intron	Nonsplice Site			G	C
MRS2085886	63.651989		Intron	Nonsplice Site			T	G
MRS2085887	63.652164		Intron	Nonsplice Site			C	G
wt37-7-63654988	63.654988	0.663	Intron	Nonsplice Site			T	C
wt37-7-63657831	63.657831	0.927	Intron	Nonsplice Site			G	A
wt37-7-63657940	63.65794	0.927	Intron	Nonsplice Site			G	A
wt37-7-63658492	63.658492	0.927	Intron	Nonsplice Site			T	C
wt37-7-63659961	63.659961	0.409	Intron	Nonsplice Site			G	A
rs31388052	63.660108		Intron	Nonsplice Site			C	A
wt37-7-63660113	63.660113	0.409	Intron	Nonsplice Site			C	A
wt37-7-63661481	63.661481		Intron	Nonsplice Site			T	C
wt37-7-63663028	63.663028	0.888	Intron	Nonsplice Site			C	T
wt37-7-63663029	63.663029	0.888	Intron	Nonsplice Site			C	T
wt37-7-63663649	63.663649	0.888	Intron	Nonsplice Site			C	T
wt37-7-63665387	63.665387	0.787	Intron	Nonsplice Site			T	C
wt37-7-63666251	63.666251	0.696	Intron	Nonsplice Site			C	T
wt37-7-63666324	63.666324	0.696	Intron	Nonsplice Site			A	T
wt37-7-63666784	63.666784	0.708	Intron	Nonsplice Site			T	C
wt37-7-63667302	63.667302	0.708	Intron	Nonsplice Site			T	C
wt37-7-63668964	63.668964	1	Intron	Nonsplice Site			A	C
wt37-7-63669068	63.669068	1	Intron	Nonsplice Site			T	A
wt37-7-63669522	63.669522	1	Intron	Nonsplice Site			A	G
wt37-7-63670458	63.670458	1	Intron	Nonsplice Site			A	G
wt37-7-63670548	63.670548	1	Intron	Nonsplice Site			C	T
wt37-7-63671117	63.671117	0.91	Intron	Nonsplice Site			G	A

wt37-7-63671275	63.671275	0.91	Intron	Nonsplice Site			C	T
wt37-7-63671397	63.671397	0.91	Intron	Nonsplice Site			G	A
wt37-7-63671627	63.671627		Intron	Nonsplice Site			T	C
wt37-7-63671628	63.671628		Intron	Nonsplice Site			T	C
wt37-7-63671695	63.671695		Intron	Nonsplice Site			C	T
wt37-7-63671754	63.671754		Intron	Nonsplice Site			T	C
wt37-7-63672467	63.672467	0.942	Intron	Nonsplice Site			G	A
wt37-7-63672571	63.672571	0.942	Intron	Nonsplice Site			T	C
wt37-7-63673787	63.673787	0.947	Intron	Nonsplice Site			C	T
wt37-7-63673855	63.673855	0.947	Intron	Nonsplice Site			A	T
wt37-7-63673903	63.673903	0.947	Intron	Nonsplice Site			T	C
wt37-7-63674065	63.674065	0.947	Intron	Nonsplice Site			A	T
wt37-7-63674070	63.67407	0.947	Intron	Nonsplice Site			A	G
wt37-7-63674076	63.674076	0.947	Intron	Nonsplice Site			T	C
wt37-7-63674083	63.674083	0.947	Intron	Nonsplice Site			T	C
wt37-7-63674220	63.67422	0.947	Intron	Nonsplice Site			T	C
wt37-7-63674254	63.674254	0.947	Intron	Nonsplice Site			T	C
wt37-7-63674363	63.674363	0.947	Intron	Nonsplice Site			C	A
MRS2085924	63.674399	0.947	Intron	Nonsplice Site			T	G
wt37-7-63674450	63.67445	0.947	Intron	Nonsplice Site			T	C
wt37-7-63674514	63.674514	1	Intron	Nonsplice Site			T	C
MRS2085927	63.674762	1	Intron	Nonsplice Site			C	T
wt37-7-63675050	63.67505	1	Intron	Nonsplice Site			G	A
wt37-7-63675203	63.675203	1	Intron	Nonsplice Site			C	A
wt37-7-63675304	63.675304	1	Intron	Nonsplice Site			A	G
wt37-7-63675460	63.67546	1	Intron	Nonsplice Site			T	C
wt37-7-63675710	63.67571	0.189	Intron	Nonsplice Site			T	C

wt37-7-63676383	63.676383	0.859	Intron	Nonsplice Site				C	T
wt37-7-63676441	63.676441	0.859	Intron	Nonsplice Site				G	T
wt37-7-63676478	63.676478	0.859	Intron	Nonsplice Site				G	A
wt37-7-63676511	63.676511	0.859	Intron	Nonsplice Site				T	G
wt37-7-63677007	63.677007	0.859	Intron	Nonsplice Site				A	G
wt37-7-63677155	63.677155		Intron	Nonsplice Site				A	G
wt37-7-63677206	63.677206		Intron	Nonsplice Site				G	A
wt37-7-63677210	63.67721		Intron	Nonsplice Site				G	A
wt37-7-63677355	63.677355	0.296	Intron	Nonsplice Site				C	T
wt37-7-63677760	63.67776		Intron	Nonsplice Site				C	G
wt37-7-63677791	63.677791		Intron	Nonsplice Site				A	G
MRS2085941	63.678092		Intron	Nonsplice Site				G	A
wt37-7-63678461	63.678461	1	Intron	Nonsplice Site				G	A
wt37-7-63678472	63.678472	1	Intron	Nonsplice Site				T	G
wt37-7-63678584	63.678584	1	Intron	Nonsplice Site				T	C
wt37-7-63678764	63.678764	1	Intron	Nonsplice Site				G	A
wt37-7-63678824	63.678824	1	Intron	Nonsplice Site				A	C
wt37-7-63678992	63.678992	1	Intron	Nonsplice Site				A	C
rs32980171	63.679138		Intron	Nonsplice Site				G	A
wt37-7-63679151	63.679151	1	Intron	Nonsplice Site				G	A
wt37-7-63679177	63.679177	1	Intron	Nonsplice Site				A	G
wt37-7-63679245	63.679245	0.979	Intron	Nonsplice Site				G	A
wt37-7-63679674	63.679674	0.979	Intron	Nonsplice Site				T	C
wt37-7-63680169	63.680169	0.979	Intron	Nonsplice Site				T	C
wt37-7-63680545	63.680545	0.465	Intron	Nonsplice Site				A	G
wt37-7-63680766	63.680766	0.465	Intron	Nonsplice Site				A	T
wt37-7-63680838	63.680838	0.465	Intron	Nonsplice Site				G	T

wt37-7-63681352	63.681352	0.565	Intron	Nonsplice Site			T	C
wt37-7-63682507	63.682507		Intron	Nonsplice Site			A	C
MRS2085957	63.682801		Intron	Nonsplice Site			A	C
wt37-7-63683682	63.683682	0.377	Intron	Nonsplice Site			A	G
wt37-7-63684421	63.684421	0.377	Intron	Nonsplice Site			A	C
wt37-7-63684738	63.684738	1	Exon 23	Coding	Nonsynonymous	Biotype: Protein Coding, K -> Q, Aag -> Cag, 794	A	C
wt37-7-63685237	63.685237	1	Intron	Nonsplice Site			C	T
wt37-7-63685702	63.685702	0.374	Intron	Nonsplice Site			G	T
wt37-7-63686055	63.686055	0.374	Intron	Nonsplice Site			C	T
wt37-7-63686329	63.686329	0.374	Intron	Nonsplice Site			G	A
wt37-7-63686513	63.686513	0.374	Intron	Nonsplice Site			T	A
wt37-7-63686669	63.686669		Intron	Nonsplice Site			C	T
wt37-7-63687254	63.687254		Intron	Nonsplice Site			G	A
wt37-7-63687744	63.687744	0.643	Intron	Nonsplice Site			A	C
wt37-7-63687745	63.687745	0.643	Intron	Nonsplice Site			A	C
wt37-7-63688029	63.688029	0.643	Intron	Nonsplice Site			G	A
wt37-7-63689473	63.689473	0.355	Intron	Nonsplice Site			C	T
wt37-7-63690599	63.690599	0.679	Intron	Nonsplice Site			C	T
wt37-7-63690617	63.690617	0.679	Intron	Nonsplice Site			T	A
MRS2085974	63.693302		Intron	Nonsplice Site			G	T
MRS2085975	63.693343		Intron	Nonsplice Site			G	C
wt37-7-63694002	63.694002		Intron	Nonsplice Site			C	T
MRS2085977	63.694412	0.831	Intron	Nonsplice Site			G	C
MRS2085978	63.69681	0.632	Intron	Nonsplice Site			C	T
wt37-7-63697901	63.697901	0.818	Intron	Nonsplice Site			T	C
wt37-7-63698958	63.698958	0.847	Intron	Nonsplice Site			A	C
wt37-7-63699757	63.699757		Intron	Nonsplice Site			A	G

wt37-7-63699764	63.699764		Intron	Nonsplice Site			A	G
MRS2085982	63.699785		Intron	Nonsplice Site			G	A
wt37-7-63699914	63.699914	0.497	Intron	Nonsplice Site			T	G
wt37-7-63700049	63.700049	0.497	Intron	Nonsplice Site			C	A
wt37-7-63700123	63.700123	0.497	Intron	Nonsplice Site			T	C
wt37-7-63700222	63.700222	0.497	Intron	Nonsplice Site			A	G
wt37-7-63700263	63.700263	0.497	Intron	Nonsplice Site			T	C
wt37-7-63700289	63.700289	0.497	Intron	Nonsplice Site			A	G
wt37-7-63700933	63.700933	0.666	Intron	Nonsplice Site			C	T
wt37-7-63701083	63.701083	0.666	Intron	Nonsplice Site			T	G
wt37-7-63701103	63.701103	0.666	Intron	Nonsplice Site			A	G
wt37-7-63701116	63.701116	0.666	Intron	Nonsplice Site			C	T
MRS2085992	63.701204		Intron	Nonsplice Site			T	C
MRS2085993	63.701208		Intron	Nonsplice Site			T	C
wt37-7-63701298	63.701298		Intron	Nonsplice Site			T	C
wt37-7-63701302	63.701302		Intron	Nonsplice Site			C	T
wt37-7-63701372	63.701372		Intron	Nonsplice Site			G	T
wt37-7-63701524	63.701524		Intron	Nonsplice Site			G	A
wt37-7-63701591	63.701591		Intron	Nonsplice Site			C	T
wt37-7-63701682	63.701682		Intron	Nonsplice Site			T	C
wt37-7-63701890	63.70189		Intron	Nonsplice Site			T	A
wt37-7-63702029	63.702029		Intron	Nonsplice Site			A	C
wt37-7-63702030	63.70203		Intron	Nonsplice Site			T	C
wt37-7-63702085	63.702085		Intron	Nonsplice Site			C	A
wt37-7-63702086	63.702086		Intron	Nonsplice Site			C	T
wt37-7-63702228	63.702228		Intron	Nonsplice Site			T	A
wt37-7-63702920	63.70292		Intron	Nonsplice Site			A	C

MRS2086003	63.703062		Intron	Nonsplice Site			T	C
MRS2086004	63.703076		Intron	Nonsplice Site			A	G
MRS2086005	63.703088		Intron	Nonsplice Site			G	C
MRS2086006	63.703176		Intron	Nonsplice Site			G	T
wt37-7-63703375	63.703375		Intron	Nonsplice Site			A	G
wt37-7-63703394	63.703394		Intron	Nonsplice Site			G	T
MRS2086010	63.70351		Intron	Nonsplice Site			A	T
MRS2086011	63.70369		Intron	Nonsplice Site			T	C
MRS2086012	63.70401		Intron	Nonsplice Site			C	T
wt37-7-63704068	63.704068		Intron	Nonsplice Site			A	T
wt37-7-63704265	63.704265		Intron	Nonsplice Site			T	A
wt37-7-63704270	63.70427		Intron	Nonsplice Site			A	T
MRS2086014	63.704337		Intron	Nonsplice Site			A	C
rs31079026	63.704505		Intron	Nonsplice Site			G	C
rs31150144	63.704506		Intron	Nonsplice Site			G	T
MRS2086015	63.704625		Intron	Nonsplice Site			T	A
MRS2086016	63.704727		Intron	Nonsplice Site			G	A
MRS2086017	63.704731		Intron	Nonsplice Site			C	A
MRS2086018	63.705098		Intron	Nonsplice Site			C	T
MRS2086019	63.705371		Intron	Nonsplice Site			T	G
MRS2086020	63.705433		Intron	Nonsplice Site			C	G
MRS2086021	63.705482		Intron	Nonsplice Site			G	T
wt37-7-63706105	63.706105		Intron	Nonsplice Site			T	C
wt37-7-63706132	63.706132		Intron	Nonsplice Site			A	C
wt37-7-63706179	63.706179		Intron	Nonsplice Site			T	C
wt37-7-63706190	63.70619		Intron	Nonsplice Site			T	C
wt37-7-63706214	63.706214		Intron	Nonsplice Site			T	C

wt37-7-63706256	63.706256		Intron	Nonsplice Site			G	C
wt37-7-63706336	63.706336		Intron	Nonsplice Site			G	A
wt37-7-63706344	63.706344		Intron	Nonsplice Site			A	G
wt37-7-63706647	63.706647		Intron	Nonsplice Site			G	A
wt37-7-63706724	63.706724		Intron	Nonsplice Site			C	T
wt37-7-63706761	63.706761		Intron	Nonsplice Site			G	A
wt37-7-63706775	63.706775		Intron	Nonsplice Site			T	G
wt37-7-63706790	63.70679		Intron	Nonsplice Site			G	A
wt37-7-63706852	63.706852		Intron	Nonsplice Site			T	C
wt37-7-63706897	63.706897		Intron	Nonsplice Site			G	C
wt37-7-63707051	63.707051		Intron	Nonsplice Site			T	A
wt37-7-63707093	63.707093		Intron	Nonsplice Site			T	A
wt37-7-63707425	63.707425		Intron	Nonsplice Site			G	A
wt37-7-63707481	63.707481		Intron	Nonsplice Site			C	A
wt37-7-63707518	63.707518		Intron	Nonsplice Site			G	T
wt37-7-63707577	63.707577		Intron	Nonsplice Site			A	T
wt37-7-63707634	63.707634		Intron	Nonsplice Site			C	T
wt37-7-63707660	63.70766		Intron	Nonsplice Site			T	C
wt37-7-63707702	63.707702		Intron	Nonsplice Site			T	A
wt37-7-63707819	63.707819		Intron	Nonsplice Site			C	T
MRS2086044	63.707853		Intron	Nonsplice Site			T	C
wt37-7-63707955	63.707955		Intron	Nonsplice Site			A	G
wt37-7-63708020	63.70802		Intron	Nonsplice Site			A	G
wt37-7-63708079	63.708079		Intron	Nonsplice Site			C	T
wt37-7-63708082	63.708082		Intron	Nonsplice Site			T	C
wt37-7-63708086	63.708086		Intron	Nonsplice Site			T	C
wt37-7-63708090	63.70809		Intron	Nonsplice Site			T	C

wt37-7-63708131	63.708131		Intron	Nonsplice Site			C	T
wt37-7-63708217	63.708217		Intron	Nonsplice Site			C	T
wt37-7-63708234	63.708234		Intron	Nonsplice Site			C	T
wt37-7-63708238	63.708238		Intron	Nonsplice Site			C	T
wt37-7-63708305	63.708305		Intron	Nonsplice Site			T	G
wt37-7-63708381	63.708381		Intron	Nonsplice Site			C	A
wt37-7-63708395	63.708395		Intron	Nonsplice Site			G	A
wt37-7-63708418	63.708418		Intron	Nonsplice Site			T	G
MRS2086052	63.708483		Intron	Nonsplice Site			G	A
wt37-7-63708487	63.708487		Intron	Nonsplice Site			G	A
wt37-7-63708499	63.708499		Intron	Nonsplice Site			A	G
wt37-7-63708551	63.708551		Intron	Nonsplice Site			C	A
wt37-7-63708730	63.70873		Intron	Nonsplice Site			G	A
wt37-7-63708776	63.708776		Intron	Nonsplice Site			T	G
wt37-7-63708862	63.708862		Intron	Nonsplice Site			C	G
wt37-7-63708903	63.708903		Intron	Nonsplice Site			T	C
wt37-7-63708989	63.708989		Intron	Nonsplice Site			A	T
wt37-7-63708997	63.708997		Intron	Nonsplice Site			T	A
wt37-7-63709049	63.709049		Intron	Nonsplice Site			C	A
wt37-7-63709073	63.709073		Intron	Nonsplice Site			T	C
wt37-7-63709133	63.709133		Intron	Nonsplice Site			A	G
wt37-7-63709140	63.70914		Intron	Nonsplice Site			T	C
wt37-7-63709177	63.709177		Intron	Nonsplice Site			T	C
wt37-7-63709244	63.709244	0.158	Intron	Nonsplice Site			C	T
wt37-7-63709298	63.709298	0.158	Intron	Nonsplice Site			C	T
wt37-7-63709300	63.7093	0.158	Intron	Nonsplice Site			G	T
wt37-7-63709439	63.709439	0.158	Intron	Nonsplice Site			A	G

wt37-7-63709454	63.709454	0.158	Intron	Nonsplice Site			G	A
wt37-7-63709537	63.709537	0.158	Intron	Nonsplice Site			T	G
MRS2086072	63.709652	0.158	Intron	Nonsplice Site			T	C
wt37-7-63709657	63.709657	0.158	Intron	Nonsplice Site			T	C
wt37-7-63709736	63.709736	0.158	Intron	Nonsplice Site			T	C
wt37-7-63709766	63.709766	0.158	Intron	Nonsplice Site			G	A
wt37-7-63709777	63.709777		Intron	Nonsplice Site			A	G
wt37-7-63709847	63.709847		Intron	Nonsplice Site			T	A
MRS2086077	63.709865		Intron	Nonsplice Site			G	T
wt37-7-63709874	63.709874		Intron	Nonsplice Site			G	T
wt37-7-63709889	63.709889		Intron	Nonsplice Site			G	A
wt37-7-63709985	63.709985	0.36	Intron	Nonsplice Site			T	C
MRS2086080	63.709989	0.36	Intron	Nonsplice Site			T	A
wt37-7-63710005	63.710005	0.36	Intron	Nonsplice Site			G	A
wt37-7-63710057	63.710057	0.36	Intron	Nonsplice Site			T	A
wt37-7-63710090	63.71009	0.36	Intron	Nonsplice Site			G	C
wt37-7-63710180	63.71018	0.36	Intron	Nonsplice Site			C	T
wt37-7-63710242	63.710242	0.36	Intron	Nonsplice Site			C	T
wt37-7-63710259	63.710259	0.36	Intron	Nonsplice Site			C	T
wt37-7-63710307	63.710307	0.36	Intron	Nonsplice Site			T	C
wt37-7-63710368	63.710368	0.36	Intron	Nonsplice Site			A	G
wt37-7-63710401	63.710401	0.36	Intron	Nonsplice Site			T	C
wt37-7-63710438	63.710438	0.36	Intron	Nonsplice Site			G	A
wt37-7-63710568	63.710568	0.36	Intron	Nonsplice Site			C	T
wt37-7-63710738	63.710738	0.36	Intron	Nonsplice Site			G	A
MRS2086093	63.710873		Intron	Nonsplice Site			T	C
wt37-7-63710941	63.710941		Intron	Nonsplice Site			T	A

wt37-7-63711215	63.711215		Intron	Nonsplice Site			T	C
wt37-7-63712393	63.712393	0.791	Intron	Nonsplice Site			G	A
wt37-7-63712410	63.71241	0.791	Intron	Nonsplice Site			G	T
wt37-7-63713522	63.713522		Intron	Nonsplice Site			C	A
wt37-7-63714550	63.71455	0.975	Intron	Nonsplice Site			T	A
wt37-7-63715518	63.715518	0.284	Intron	Nonsplice Site			A	G
rs32357610	63.715719		Intron	Nonsplice Site			A	T
wt37-7-63717473	63.717473	0.972	Intron	Nonsplice Site			C	T
wt37-7-63717651	63.717651	0.972	Intron	Nonsplice Site			C	T
wt37-7-63717829	63.717829	0.972	Intron	Nonsplice Site			T	A
wt37-7-63718319	63.718319		Intron	Nonsplice Site			T	A
wt37-7-63718477	63.718477		Intron	Nonsplice Site			G	T
wt37-7-63718604	63.718604		Intron	Nonsplice Site			C	A
wt37-7-63719189	63.719189	0.464	Intron	Nonsplice Site			T	G
rs32981636	63.719306		Intron	Nonsplice Site			A	G
wt37-7-63719500	63.7195	0.464	Intron	Nonsplice Site			G	T
wt37-7-63719820	63.71982	0.464	Intron	Nonsplice Site			G	A
wt37-7-63719872	63.719872		Intron	Nonsplice Site			A	G
wt37-7-63720092	63.720092		Intron	Nonsplice Site			C	A
wt37-7-63720244	63.720244		Intron	Nonsplice Site			G	T
wt37-7-63720568	63.720568	0.523	Intron	Nonsplice Site			T	C
wt37-7-63720812	63.720812	0.523	Intron	Nonsplice Site			A	T
wt37-7-63721085	63.721085		Intron	Nonsplice Site			T	G
wt37-7-63721091	63.721091		Intron	Nonsplice Site			T	G
MRS2086110	63.721199		Intron	Nonsplice Site			C	T
MRS2086111	63.72161		Intron	Nonsplice Site			A	T
MRS2086112	63.721697		Intron	Nonsplice Site			C	T

MRS2086113	63.722132		Intron	Nonsplice Site			T	G
MRS2086114	63.722135		Intron	Nonsplice Site			G	T
MRS2086115	63.722144		Intron	Nonsplice Site			G	T
MRS2086116	63.722276		Intron	Nonsplice Site			G	T
MRS2086117	63.722423		Intron	Nonsplice Site			C	T
MRS2086119	63.724042		Intron	Nonsplice Site			C	T
MRS2086120	63.724199		Intron	Nonsplice Site			A	G
MRS2086121	63.72433		Intron	Nonsplice Site			A	G
MRS2086122	63.724641		Intron	Nonsplice Site			G	A
wt37-7-63724831	63.724831		Intron	Nonsplice Site			G	A
wt37-7-63724919	63.724919		Intron	Nonsplice Site			G	A
wt37-7-63725533	63.725533	0.739	Intron	Nonsplice Site			A	G
wt37-7-63725593	63.725593	0.739	Intron	Nonsplice Site			C	T
wt37-7-63725677	63.725677	0.739	Intron	Nonsplice Site			C	T
wt37-7-63725839	63.725839	0.739	Intron	Nonsplice Site			T	C
wt37-7-63725945	63.725945	0.739	Intron	Nonsplice Site			G	A
wt37-7-63726135	63.726135	0.739	Intron	Nonsplice Site			T	G
wt37-7-63726168	63.726168	0.405	Intron	Nonsplice Site			G	A
wt37-7-63726838	63.726838	0.554	Intron	Nonsplice Site			T	C
wt37-7-63727063	63.727063	0.554	Intron	Nonsplice Site			T	C
wt37-7-63727560	63.72756	0.554	Intron	Nonsplice Site			A	G
wt37-7-63727838	63.727838	0.779	Intron	Nonsplice Site			A	G
wt37-7-63727918	63.727918	0.779	Intron	Nonsplice Site			C	T
wt37-7-63728017	63.728017	0.779	Intron	Nonsplice Site			G	T
wt37-7-63728267	63.728267	0.779	Intron	Nonsplice Site			C	T
wt37-7-63728323	63.728323	0.779	Intron	Nonsplice Site			C	G
wt37-7-63728342	63.728342	0.779	Intron	Nonsplice Site			A	G

wt37-7-63728597	63.728597	0.779	Intron	Nonsplice Site			G	A
wt37-7-63729138	63.729138	0.517	Intron	Nonsplice Site			T	A
wt37-7-63729391	63.729391	0.517	Intron	Nonsplice Site			T	A
wt37-7-63729948	63.729948	0.659	Intron	Nonsplice Site			G	A
wt37-7-63730674	63.730674	0.659	Intron	Nonsplice Site			C	T
wt37-7-63731027	63.731027	0.685	Intron	Nonsplice Site			G	A
wt37-7-63731713	63.731713	0.685	Intron	Nonsplice Site			C	T
wt37-7-63731794	63.731794	0.685	Intron	Nonsplice Site			A	G
wt37-7-63731964	63.731964	0.086	Intron	Nonsplice Site			T	C
wt37-7-63732094	63.732094	0.086	Intron	Nonsplice Site			C	T
wt37-7-63732142	63.732142	0.086	Intron	Nonsplice Site			C	T
wt37-7-63732367	63.732367	0.086	Intron	Nonsplice Site			G	C
wt37-7-63732389	63.732389	0.086	Intron	Nonsplice Site			T	C
wt37-7-63732737	63.732737	0.086	Intron	Nonsplice Site			A	G
wt37-7-63732788	63.732788		Intron	Nonsplice Site			T	A
wt37-7-63732789	63.732789		Intron	Nonsplice Site			T	A
wt37-7-63732796	63.732796		Intron	Nonsplice Site			T	A
wt37-7-63732816	63.732816		Intron	Nonsplice Site			T	G
wt37-7-63732829	63.732829		Intron	Nonsplice Site			T	C
wt37-7-63732834	63.732834		Intron	Nonsplice Site			A	T
MRS2086152	63.732848		Intron	Nonsplice Site			C	T
MRS2086153	63.732858		Intron	Nonsplice Site			C	T
wt37-7-63733837	63.733837		Intron	Nonsplice Site			C	T
wt37-7-63733849	63.733849		Intron	Nonsplice Site			C	T
wt37-7-63733850	63.73385		Intron	Nonsplice Site			A	G
wt37-7-63733890	63.73389	0.995	Intron	Nonsplice Site			A	G
wt37-7-63734081	63.734081	0.995	Intron	Nonsplice Site			T	C

wt37-7-63734425	63.734425	0.995	Intron	Nonsplice Site			C	G
wt37-7-63734456	63.734456	0.995	Intron	Nonsplice Site			G	A
wt37-7-63734530	63.73453	0.995	Intron	Nonsplice Site			G	T
wt37-7-63734556	63.734556	0.995	Intron	Nonsplice Site			G	A
wt37-7-63734565	63.734565	0.995	Intron	Nonsplice Site			C	A
wt37-7-63735003	63.735003		Intron	Nonsplice Site			T	A
wt37-7-63735060	63.73506		Intron	Nonsplice Site			G	T
MRS2086165	63.735249		Intron	Nonsplice Site			A	T
MRS2086166	63.735296		Intron	Nonsplice Site			C	A
MRS2086167	63.735317		Intron	Nonsplice Site			A	G
MRS2086168	63.73534		Intron	Nonsplice Site			A	G
MRS2086169	63.735354		Intron	Nonsplice Site			C	T
MRS2086170	63.735398		Intron	Nonsplice Site			C	A
MRS2086171	63.735436		Intron	Nonsplice Site			G	A
MRS2086172	63.735538		Intron	Nonsplice Site			G	T
wt37-7-63736028	63.736028	0.488	Intron	Nonsplice Site			G	C
wt37-7-63736147	63.736147	0.488	Intron	Nonsplice Site			A	G
wt37-7-63736149	63.736149	0.488	Intron	Nonsplice Site			A	G
wt37-7-63736322	63.736322		Intron	Nonsplice Site			G	T
wt37-7-63736609	63.736609		Intron	Nonsplice Site			C	T
wt37-7-63736677	63.736677		Intron	Nonsplice Site			T	C
MRS2086177	63.736695		Intron	Nonsplice Site			C	G
wt37-7-63737200	63.7372		Intron	Nonsplice Site			T	C
wt37-7-63738817	63.738817		Intron	Nonsplice Site			G	A
wt37-7-63738856	63.738856		Intron	Nonsplice Site			A	G
wt37-7-63739035	63.739035		Intron	Nonsplice Site			T	G
wt37-7-63739556	63.739556		Intron	Nonsplice Site			A	G

wt37-7-63741564	63.741564	0.351	Intron	Nonsplice Site			C	T
rs32093890	63.742094		Intron	Nonsplice Site			C	T
wt37-7-63742106	63.742106		Intron	Nonsplice Site			C	T
wt37-7-63742113	63.742113		Intron	Nonsplice Site			T	C
wt37-7-63742678	63.742678	0.625	Intron	Nonsplice Site			T	C
wt37-7-63742995	63.742995		Intron	Nonsplice Site			G	A
wt37-7-63743229	63.743229		Intron	Nonsplice Site			T	G
MRS2086187	63.743303		Intron	Nonsplice Site			T	G
wt37-7-63743355	63.743355		Intron	Nonsplice Site			A	G
wt37-7-63744467	63.744467	0.915	Intron	Nonsplice Site			C	T
wt37-7-63745102	63.745102	0.612	Intron	Nonsplice Site			G	C
wt37-7-63746954	63.746954		Intron	Nonsplice Site			T	G
wt37-7-63747672	63.747672		Intron	Nonsplice Site			C	T
wt37-7-63748222	63.748222		Intron	Nonsplice Site			G	A
MRS2086194	63.750273		Intron	Nonsplice Site			G	A
wt37-7-63751746	63.751746		Intron	Nonsplice Site			C	A
wt37-7-63752812	63.752812	0.745	Intron	Nonsplice Site			C	T
wt37-7-63755315	63.755315	0.97	Intron	Nonsplice Site			G	C
wt37-7-63756180	63.75618	0.985	Intron	Nonsplice Site			G	T
wt37-7-63756647	63.756647	0.985	Intron	Nonsplice Site			C	T
rs31807960	63.757949		Intron	Nonsplice Site			T	G
rs31822218	63.757952		Intron	Nonsplice Site			T	G
wt37-7-63758147	63.758147	0.735	Intron	Nonsplice Site			A	T
wt37-7-63758400	63.7584	0.735	Intron	Nonsplice Site			A	G
rs32160858	63.759282		Intron	Nonsplice Site			T	A
rs32988094	63.759958		Intron	Nonsplice Site			A	T
wt37-7-63760527	63.760527	0.667	Intron	Nonsplice Site			A	G

MRS2086203	63.765178	0.374	Intron	Nonsplice Site			C	A
wt37-7-63766747	63.766747	0.855	Intron	Nonsplice Site			A	G
wt37-7-63774037	63.774037	0.515	Intron	Nonsplice Site			A	G
MRS2086207	63.774264	0.515	Intron	Nonsplice Site			G	A
wt37-7-63774866	63.774866	0.8	Intron	Nonsplice Site			C	A
wt37-7-63775428	63.775428	0.36	Intron	Nonsplice Site			C	T
wt37-7-63776055	63.776055	0.36	Intron	Nonsplice Site			A	G
wt37-7-63776236	63.776236	0.36	Intron	Nonsplice Site			A	T
wt37-7-63777544	63.777544		Intron	Nonsplice Site			G	T
wt37-7-63778769	63.778769	0.522	Intron	Nonsplice Site			G	A
wt37-7-63779192	63.779192	0.522	Intron	Nonsplice Site			G	A
wt37-7-63779525	63.779525	0.522	Intron	Nonsplice Site			A	C
MRS2086216	63.781514	0.814	Intron	Nonsplice Site			G	T
wt37-7-63783402	63.783402	0.798	Intron	Nonsplice Site			T	A
wt37-7-63784260	63.78426		Intron	Nonsplice Site			G	A
wt37-7-63785970	63.78597		Intron	Nonsplice Site			A	G
wt37-7-63786678	63.786678		Intron	Nonsplice Site			C	T
MRS2086222	63.787416		Intron	Nonsplice Site			C	T
wt37-7-63788078	63.788078		Intron	Nonsplice Site			T	C
wt37-7-63788210	63.78821		Intron	Nonsplice Site			T	A
wt37-7-63788643	63.788643		Intron	Nonsplice Site			T	C
wt37-7-63788967	63.788967	0.747	Intron	Nonsplice Site			T	G
MRS2086227	63.788968	0.747	Intron	Nonsplice Site			G	T
MRS2086228	63.788969	0.747	Intron	Nonsplice Site			T	G
wt37-7-63791328	63.791328	1	Exon 24	Coding	Nonsynonymous	Biotype: Protein Coding, V -> M, Gtg -> Atg, 811	G	A
wt37-7-63791511	63.791511	1	Exon	3' UTR			C	T
