# Supplementary material for: Genetic modulation of the iris transillumination defect: a systems genetics analysis using the expanded family of BXD glaucoma strains
Source: Pigment Cell Melanoma Res. 2013 Apr 13;26(4):487–98. doi: 10.1111/pcmr.12106 (PMC3752936; doi:10.1111/pcmr.12106)
Supplement: Supplementary file 5 [file pcmr0026-0487-SD5.pdf]

Appendix 5: List of SNPs within *Myo5a*

SNP ID	Mb	ConScore	Domain 1	Domain 2	Function	Details	B6	D2
wt37-9-74928974	74.928974	0.82	Intron	Nonsplice Site			C	T
MRS2755623	74.93459		Intron	Nonsplice Site			C	A
MRS2755624	74.962261		Intron	Nonsplice Site			G	A
wt37-9-74970087	74.970087	1	Intron	Nonsplice Site			A	G
wt37-9-74973473	74.973473	0.776	Intron	Nonsplice Site			A	G
wt37-9-74974134	74.974134	0.972	Intron	Nonsplice Site			A	G
rs32945910	74.979146		Intron	Nonsplice Site			A	G
wt37-9-74982939	74.982939	0.923	Intron	Nonsplice Site			G	A
wt37-9-74985238	74.985238	0.992	Intron	Nonsplice Site			C	A
wt37-9-74992337	74.992337	1	Intron	Nonsplice Site			G	A
wt37-9-74993105	74.993105	0.586	Intron	Nonsplice Site			C	T
wt37-9-75013566	75.013566	1	Intron	Nonsplice Site			T	C
wt37-9-75016258	75.016258	1	Intron	Nonsplice Site			T	A
wt37-9-75026248	75.026248	0.491	Intron	Nonsplice Site			A	T
wt37-9-75034469	75.034469	1	Intron	Nonsplice Site			T	C
MRS2755637	75.035358	1	Intron	Nonsplice Site			T	G
MRS2755638	75.037585	1	Intron	Nonsplice Site			T	C
wt37-9-75039662	75.039662		Intron	Nonsplice Site			C	A
wt37-9-75039783	75.039783	1	Intron	Nonsplice Site			G	C
wt37-9-75050742	75.050742	0.677	Intron	Nonsplice Site			T	C
wt37-9-75053151	75.053151	0.855	Intron	Nonsplice Site			T	A
wt37-9-75053815	75.053815	0.92	Intron	Nonsplice Site			C	T
wt37-9-75061676	75.061676	0.475	Intron	Nonsplice Site			C	T
MRS2755645	75.061932	0.475	Intron	Nonsplice Site			G	A
wt37-9-75062374	75.062374	0.475	Intron	Nonsplice Site			T	C
