# Supplementary material for: Genetic modulation of the iris transillumination defect: a systems genetics analysis using the expanded family of BXD glaucoma strains
Source: Pigment Cell Melanoma Res. 2013 Apr 13;26(4):487–98. doi: 10.1111/pcmr.12106 (PMC3752936; doi:10.1111/pcmr.12106)
Supplement: Supplementary file 6 [file pcmr0026-0487-SD6.pdf]

Appendix 6: List of SNPs within *Prkcz*

SNP ID	Mb	ConScore	Domain 1	Domain 2	Function	Details	B6	D2
wt37-4-154634448	154.634448	1	Exon	3' UTR				C
rs33152187	154.647859		Intron	Nonsplice Site			G	
rs32123501	154.653871		Intron	Nonsplice Site			T	C
wt37-4-154657047	154.657047	0.213	Intron	Nonsplice Site				G
rs33146997	154.660762		Intron	Nonsplice Site				C
rs33147836	154.660979		Exon 7	Coding	Nonsynonymous	Biotype: Protein Coding, D -> V, gAc -> gTc, 112	T	
wt37-4-154709187	154.709187	0.49	Intron	Nonsplice Site			T	C
wt37-4-154713815	154.713815	0.397	Intron	Nonsplice Site				T
MRS1470012	154.713835	0.397	Intron	Nonsplice Site			C	T
rs33155545	154.714496		Intron	Nonsplice Site			C	
wt37-4-154734934	154.734934	1	Intron	Nonsplice Site				T
