# Supplementary material for: Genetic modulation of the iris transillumination defect: a systems genetics analysis using the expanded family of BXD glaucoma strains
Source: Pigment Cell Melanoma Res. 2013 Apr 13;26(4):487–98. doi: 10.1111/pcmr.12106 (PMC3752936; doi:10.1111/pcmr.12106)
Supplement: Supplementary file 7 [file pcmr0026-0487-SD7.pdf]

**Appendix 7:** List of SNPs within *Zbtb20*

| SNP ID | Mb | ConScore | Domain 1 | Domain 2 | Function | Details |
|---|---|---|---|---|---|---|
| wt37-16-43513291 | 43.513291 | 1 | Intron | Nonsplice Site | | |
| rs4179183 | 43.517049 | | Intron | Nonsplice Site | | |
| wt37-16-43517134 | 43.517134 | 1 | Intron | Nonsplice Site | | |
| wt37-16-43518640 | 43.518640 | 1 | Intron | Nonsplice Site | | |
| wt37-16-43520688 | 43.520688 | 0.216 | Intron | Nonsplice Site | | |
| wt37-16-43521726 | 43.521726 | 1 | Intron | Nonsplice Site | | |
| rs4179188 | 43.522545 | | Intron | Nonsplice Site | | |
| wt37-16-43522553 | 43.522553 | 1 | Intron | Nonsplice Site | | |
| wt37-16-43522669 | 43.522669 | 1 | Intron | Nonsplice Site | | |
| wt37-16-43522670 | 43.522670 | 1 | Intron | Nonsplice Site | | |
| wt37-16-43523914 | 43.523914 | 1 | Intron | Nonsplice Site | | |
| MRS4292163 | 43.524384 | 1 | Intron | Nonsplice Site | | |
| wt37-16-43524387 | 43.524387 | 1 | Intron | Nonsplice Site | | |
| MRS4292164 | 43.524476 | 0.743 | Intron | Nonsplice Site | | |
| wt37-16-43527211 | 43.527211 | 0.978 | Intron | Nonsplice Site | | |
| wt37-16-43529088 | 43.529088 | 0.988 | Intron | Nonsplice Site | | |
| wt37-16-43529212 | 43.529212 | 0.84 | Intron | Nonsplice Site | | |
| wt37-16-43529356 | 43.529356 | 0.84 | Intron | Nonsplice Site | | |
| wt37-16-43531082 | 43.531082 | 1 | Intron | Nonsplice Site | | |
| MRS4292170 | 43.531336 | 1 | Intron | Nonsplice Site | | |
| wt37-16-43533558 | 43.533558 | 1 | Intron | Nonsplice Site | | |
| wt37-16-43538577 | 43.538577 | 1 | Intron | Nonsplice Site | | |
| wt37-16-43540984 | 43.540984 | 1 | Intron | Nonsplice Site | | |
| wt37-16-43543216 | 43.543216 | 0.997 | Intron | Nonsplice Site | | |
| wt37-16-43546442 | 43.546442 | 1 | Intron | Nonsplice Site | | |
| wt37-16-43546456 | 43.546456 | 1 | Intron | Nonsplice Site | | |
| wt37-16-43546651 | 43.546651 | 1 | Intron | Nonsplice Site | | |

| | | | | | | |
|---|---|---|---|---|---|---|
| wt37-16-43547658 | 43.547658 | 1 | Intron | Nonsplice Site | | |
| wt37-16-43557749 | 43.557749 | 1 | Intron | Nonsplice Site | | |
| wt37-16-43558021 | 43.558021 | 1 | Intron | Nonsplice Site | | |
| wt37-16-43558022 | 43.558022 | 1 | Intron | Nonsplice Site | | |
| wt37-16-43559102 | 43.559102 | 0.889 | Intron | Nonsplice Site | | |
| wt37-16-43565706 | 43.565706 | 1 | Intron | Nonsplice Site | | |
| wt37-16-43568763 | 43.568763 | 0.646 | Intron | Nonsplice Site | | |
| wt37-16-43569718 | 43.569718 | 1 | Intron | Nonsplice Site | | |
| wt37-16-43574894 | 43.574894 | 1 | Intron | Nonsplice Site | | |
| wt37-16-43575784 | 43.575784 | 0.994 | Intron | Nonsplice Site | | |
| rs4179206 | 43.578872 | | Intron | Nonsplice Site | | |
| wt37-16-43578960 | 43.578960 | 1 | Intron | Nonsplice Site | | |
| wt37-16-43579291 | 43.579291 | 1 | Intron | Nonsplice Site | | |
| wt37-16-43580502 | 43.580502 | 1 | Intron | Nonsplice Site | | |
| wt37-16-43581016 | 43.581016 | 1 | Intron | Nonsplice Site | | |
| MRS4292191 | 43.582640 | 1 | Intron | Nonsplice Site | | |
| wt37-16-43582999 | 43.582999 | 1 | Intron | Nonsplice Site | | |
| wt37-16-43583324 | 43.583324 | 1 | Intron | Nonsplice Site | | |
| wt37-16-43584126 | 43.584126 | 1 | Intron | Nonsplice Site | | |
| wt37-16-43585477 | 43.585477 | 0.511 | Intron | Nonsplice Site | | |
| wt37-16-43585480 | 43.585480 | 0.511 | Intron | Nonsplice Site | | |
| wt37-16-43585481 | 43.585481 | 0.511 | Intron | Nonsplice Site | | |
| wt37-16-43589093 | 43.589093 | 1 | Intron | Nonsplice Site | | |
| wt37-16-43591576 | 43.591576 | 0.982 | Intron | Nonsplice Site | | |
| wt37-16-43591831 | 43.591831 | 0.982 | Intron | Nonsplice Site | | |
| wt37-16-43592084 | 43.592084 | 0.982 | Intron | Nonsplice Site | | |
| wt37-16-43592510 | 43.592510 | 0.982 | Intron | Nonsplice Site | | |
| wt37-16-43592523 | 43.592523 | 0.982 | Intron | Nonsplice Site | | |
| wt37-16-43592687 | 43.592687 | 1 | Intron | Nonsplice Site | | |

| | | | | | | |
|---|---|---|---|---|---|---|
| MRS4292204 | 43.596136 | 1 | Intron | Nonsplice Site | | |
| wt37-16-43596169 | 43.596169 | 1 | Intron | Nonsplice Site | | |
| wt37-16-43596899 | 43.596899 | 1 | Intron | Nonsplice Site | | |
| wt37-16-43597013 | 43.597013 | 1 | Intron | Nonsplice Site | | |
| wt37-16-43599237 | 43.599237 | 1 | Intron | Nonsplice Site | | |
| wt37-16-43599392 | 43.599392 | 1 | Intron | Nonsplice Site | | |
| wt37-16-43600620 | 43.600620 | 0.972 | Intron | Nonsplice Site | | |
| wt37-16-43601601 | 43.601601 | 0.972 | Intron | Nonsplice Site | | |
| wt37-16-43602212 | 43.602212 | 0.997 | Intron | Nonsplice Site | | |
| wt37-16-43602973 | 43.602973 | 0.116 | Intron | Nonsplice Site | | |
| wt37-16-43603136 | 43.603136 | 0.116 | Intron | Nonsplice Site | | |
| wt37-16-43603140 | 43.603140 | 0.116 | Intron | Nonsplice Site | | |
| wt37-16-43603191 | 43.603191 | 0.116 | Intron | Nonsplice Site | | |
| wt37-16-43605226 | 43.605226 | 1 | Intron | Nonsplice Site | | |
| wt37-16-43605334 | 43.605334 | 1 | Intron | Nonsplice Site | | |
| wt37-16-43606474 | 43.606474 | 0.201 | Intron | Nonsplice Site | | |
| MRS4292221 | 43.607257 | 0.626 | Intron | Nonsplice Site | | |
| wt37-16-43608434 | 43.608434 | 0.625 | Intron | Nonsplice Site | | |
| wt37-16-43608778 | 43.608778 | 0.625 | Intron | Nonsplice Site | | |
| wt37-16-43610176 | 43.610176 | 1 | Exon 8 | Coding | Synonymous | Biotype: Protein Coding, R -> R, cgG -> cgC, 312 |
| wt37-16-43610605 | 43.610605 | 1 | Exon 8 | Coding | Synonymous | Biotype: Protein Coding, G -> G, ggC -> ggT, 455 |
| wt37-16-43610878 | 43.610878 | 1 | Exon 8 | Coding | Synonymous | Biotype: Protein Coding, G -> G, ggT -> ggC, 546 |
| rs4179234 | 43.611431 | | Intron | Nonsplice Site | | |
| wt37-16-43611512 | 43.611512 | 0.853 | Intron | Nonsplice Site | | |
| wt37-16-43611549 | 43.611549 | 0.853 | Intron | Nonsplice Site | | |
| wt37-16-43613040 | 43.613040 | 0.894 | Intron | Nonsplice Site | | |
| wt37-16-43614116 | 43.614116 | 1 | Intron | Nonsplice Site | | |
| wt37-16-43615193 | 43.615193 | 0.877 | Intron | Nonsplice Site | | |
| wt37-16-43615268 | 43.615268 | 1 | Intron | Nonsplice Site | | |

| | | | | | | |
|---|---|---|---|---|---|---|
| wt37-16-43615301 | 43.615301 | 1 | Intron | Nonsplice Site | | |
| wt37-16-43617791 | 43.617791 | 1 | Intron | Nonsplice Site | | |
