# Supplementary material for: Genetic modulation of the iris transillumination defect: a systems genetics analysis using the expanded family of BXD glaucoma strains
Source: Pigment Cell Melanoma Res. 2013 Apr 13;26(4):487–98. doi: 10.1111/pcmr.12106 (PMC3752936; doi:10.1111/pcmr.12106)
Supplement: Supplementary file 8 [file pcmr0026-0487-SD8.pdf]

Appendix 8: Screening of known pigmentation and iris phenotype related genes

Known Pigmentation /Iris related phenotype genes	Gene Location from GeneNetwork	Comment
Tyr - tyrosinase (albino c locus, oculocutaneous albinism IA)	Chr7: 94.57 Mb	*Tyr* does not line up across various age groups in the QTL peaks as seen in Figures 5F to 5J, and the peak LRS score is very low.
Dct - dopachrome tautomerase	Chr 14: 118.41 Mb	We see a suggestive QTL for *Dct* in Figure 5J. However we excluded this candidate since it did not have any functional SNP and lacked any strong evidence for a clinically relevant phenotype. Additionally it did not possess a cis-eQTL.
Vps33a - vacuolar protein sorting 33a	Chr5: 123.98 Mb	*Vps33a* does not fall under any of the QTL peaks seen in Figures 5F to 5J.
Hps3 - Hermansky-Pudlak syndrome 3 homolog	Chr3: 19.89 Mb	*Hps3* does not fall under any of the QTL peaks seen in Figures 5F to 5J.
Rabggta - Rab geranylgeranyl transferase, a subunit	Chr14: 56.33 Mb	*Rabggta* does not fall under any of the QTL peaks seen in Figures 5F to 5J.
Mlph - melanophilin (leaden)	Chr1: 92.84 Mb	*Mlph* does not coincide with the QTLs seen in Chr 1 (Figure 5G), wherein there are peaks at 50-75 Mb, 125-150 Mb and around 175 Mb.
Hps1 - Hermansky-Pudlak syndrome 1 homolog	Chr19: 42.82 Mb	*Hps1* does not coincide with the QTLs seen in Chr 1 (Figure 5G), wherein there are peaks at 50-75 Mb, 125-150 Mb and around 175 Mb. The QTLs in Chr 19 as seen in the Figure 5F are seen at less than 25 Mb and around 30 Mb. Hence the peak for *Hps1* does not match with the QTLs seen in the Figure 5F.
Pldn - pallidin	Chr2: 122.57 Mb	*Pldn* does not coincide with the QTLs seen in Chr 2 (Figure 5F), wherein the peak is at 100 Mb.
Mc1r - melanocortin 1 receptor	Chr8: 125.93 Mb	*Mc1r* does not fall under any of the QTL peaks indicated in Figures 5F to 5J.
Myo7a - myosin VIIa	Chr7: 105.20 Mb	*Myo7a* does not fall under any of the QTL peaks indicated in Figures 5F to 5J.
Si - silver aka Pmel – premelanosome protein	Chr10: 128.15 Mb	*Si* does not fall under any of the QTL peaks indicated in Figures 5F to 5J.
Mitf - microphthalmia- associated transcription factor	Chr6: 97.94 Mb	*Mitf* does not line up across the various age groups and the peak LRS score is very low.
Slc45a2 - solute carrier family 45	Chr15: 10.96 Mb	*Slc45a2* does not fall under any of the QTL peaks indicated in

		Figures 5F to 5J.
a - nonagouti	Chr2: 154.85 Mb	*nonagouti* does not fall under any of the QTL peaks indicated in Figures 5F to 5J.
Pomc1 - pro-opiomelanocortin alpha	Chr12: 3.95 Mb	*Pomc1* does not fall under any of the QTL peaks indicated in Figures 5F to 5J.
Rab27a - member RAS oncogene family	Chr9: 72.9 Mb	Located very close to Myo5a. However *Rab27a* does not possess a cis-eQTL, hence was not considered for the current paper.
Hps6 - Hermansky-Pudlak syndrome 6	Chr19: 46.08 Mb	*Hps6* does not fall under any of the QTL peaks indicated in Figures 5F to 5J. In Chr 19, the peak seen does not line up across various age groups and neither does it have a high LRS.
Hps5 - Hermansky-Pudlak syndrome 5	Chr7: 54.01 Mb	*Hps5* is close to the QTL peak chosen in Figure 5F, however does not fulfill the inclusion criteria (SNPs or Indels absent) also no cis eQTL.
Rpl24 - ribosomal protein L24	Chr16: 55.96 Mb	*Rpl24* does not fall within ±5 Mb of the peak considered in Chr16 (38-46 Mb), as mentioned in the inclusion criteria.
Pitx3 - paired-like homeodomain transcription factor 3	Chr19: 46.21 Mb	QTL within Chr19 does not fulfill our inclusion criteria.
Dcc - deleted in colorectal carcinoma	Chr18: 71.41 Mb	QTL within Chr18 does not fulfill our inclusion criteria.
Pax6 - paired box gene 6	Chr2: 105.5 Mb	There is no QTL at this location.
Nf1 - neurofibromatosis 1	Chr11: 79.28 Mb	There is no QTL at this location.
Rbp7 - retinol binding protein 7	Chr4: 148.82 Mb	Located close to *Prkcz* within the QTl at Chr4, however does not fulfill our inclusion criteria
Nmnat1 - nicotinamide nucleotide adenylyltransferase 1	Chr4: 148.84 Mb	Located close to *Prkcz* within the QTl at Chr4, however does not fulfill our inclusion criteria
Hes3 - hairy and enhancer of split 3	Chr4: 151.66 Mb	Located close to *Prkcz* within the QTl at Chr4, however does not fulfill our inclusion criteria
Nphp4 - nephronophthisis 4	Chr4: 151.93 Mb	Located close to *Prkcz* within the QTl at Chr4, however does not fulfill our inclusion criteria
Prdm16 - PR domain containing 16	Chr4: 153.69 Mb	Located close to *Prkcz* within the QTl at Chr4, however does not fulfill our inclusion criteria
Rab27b - member RAS oncogene family	Chr18: 70.14 Mb	QTL within Chr18 does not fulfill our inclusion criteria.
Gif - gastric intrinsic factor	Chr19: 11.83 Mb	QTL within Chr19 does not fulfill our inclusion criteria.
